# Supplementary material for: Extreme longitudinal thermal conductivity and non-diffusive heat transport in isotopic hBN
Source: Nat Commun. 2026 Mar 2;17:3352. doi: 10.1038/s41467-026-69907-x (PMC13066571; doi:10.1038/s41467-026-69907-x)
Supplement: Supplementary file 1 — Supplementary Information [file 41467_2026_69907_MOESM1_ESM.pdf]

Extreme Longitudinal Thermal Conductivity and Non-Diffusive Heat Transport in Isotopic hBN

Cléophanie Brochard-Richard[1], Gaia Di Berardino[1], Etienne Herth[1], Chen Wei [1], Federico Panciera [1], Thomas Poirier[2], James H. Edgar [2,], , Bernard Gil [3], Guillaume Cassabois [3], Maria Luisa Della Rocca[4], Suman Sarkar [5], Nedjma Bendiab [5], Laëtitia Marty [5], Fabrice Oehler [1], Abdelkarim Ouerghi [1], Julien Chaste [1*]

[1]Université Paris-Saclay, CNRS, Centre de Nanosciences et de Nanotechnologies, 91120, Palaiseau, France.
2 Tim Taylor Department of Chemical Engineering, Kansas State University, Durland Hall, Manhattan, KS 66506-5102, USA
3 Laboratoire Charles Coulomb (L2C), UMR 5221 CNRS-Université de Montpellier, F-34095 Montpellier, France
4 Université Paris Cité, CNRS, Laboratoire Matériaux et Phénomènes Quantiques, F-75013, Paris, France
5 Université Grenoble Alpes, CNRS, Grenoble INP, Institut NEEL, F-38000, Grenoble, France.
* Corresponding author: julien.chaste@universite-paris-saclay.fr

Supplementary notes

1. Soft transfer method 2
2. Typical samples 3
3. Raman spectrum and temperature calibration 5
4. Joule heating, laser heating and WSe_2 thermometry 8
5. Absence of deformation and optical separation of strain from Raman measurements 9
6. Temperature uncertainty and parasitic thermal effects 11
7. Temperature dependence of the electrical and thermal conductivity for LMM1, LMM2 and LMMGr. 13
8. Simulations for LMMGR: 2-points configuration. 15
9. Simulations for LMM2: temperature dependence of k_{2D}. 16
10. Simulations for LMM2: out-of-plane thermal conductivity 17
11. Simulations for LMM1: thermal contact simulation 19
12. Thermal conductivity of hBN and graphene in the literature. 20
13. Thermoreflectance on the supported isotopic hBN 24
14. Example of spectra during the measurements (Figure 4a) 25
15. A reversed temperature bias in Figure 4e 26

1. Soft transfer method

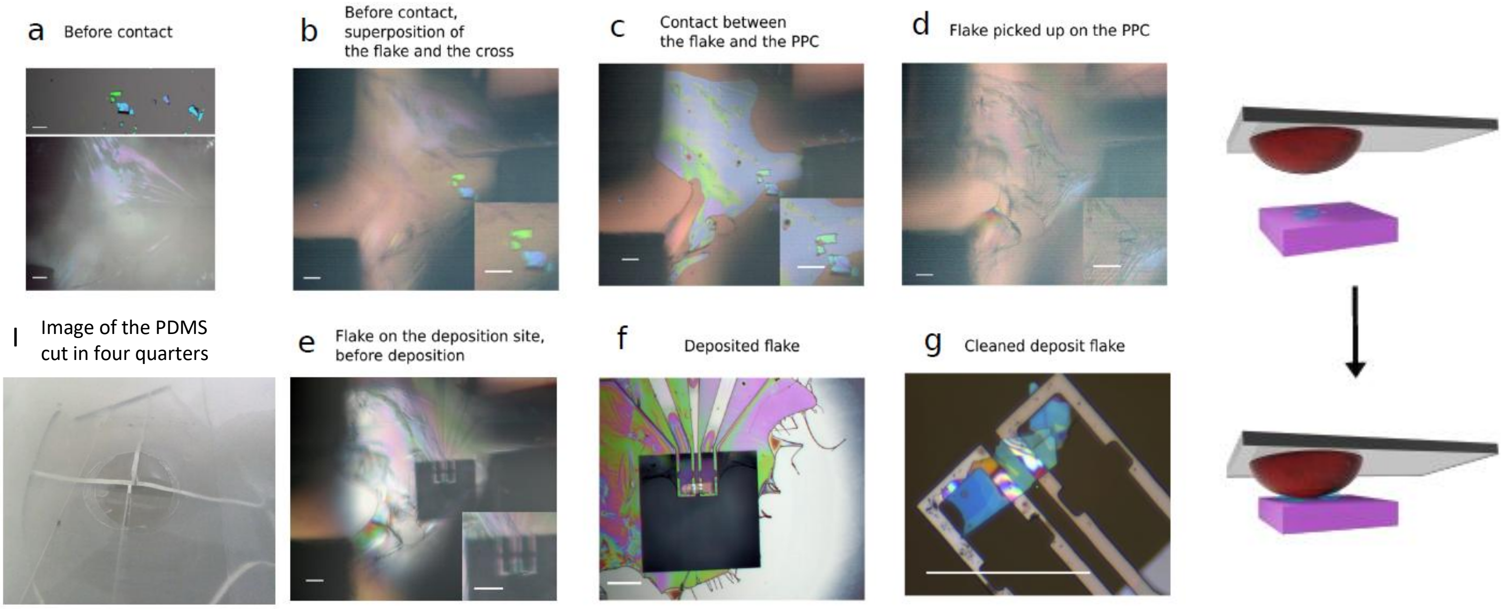

Figure S1-1: The different steps of the soft dry transfer process developed to transfer the 2D material on top of the microheater without any strain. Scale bar=50 µm. 3D image are done with Blender.

The transfers were performed using a commercial transfer station from HQ Graphene. For the exfoliated hBN, the flake was transferred using the pick-up technique [1,2], consisting of PPC-coated PDMS hemispheres, to catch the flakes. The use of water is known to enhance transfer efficiency due to capillary forces between water and two-dimensional materials [3,4], and has already been employed by authors[5–7]. This technique was employed for the transfer of WSe_2 grown by chemical vapour deposition (CVD). To collect the WSe_2 flakes, a volume of 3 µL of demineralized water, with a droplet diameter of about 400 µm was deposited to the surface. The PPC/PDMS structure is then brought into contact with the flakes. The temperature is bring to 308.15 K in order to optimize the contact. The flakes are then removed from the surface during the pick-up step at 300K.
Applying the 2D material onto the silicon cantilevers is a delicate process. In contrast to the typical fabrication of suspended 2D materials, it is essential to consider the pressure exerted by the PDMS hemisphere on the suspended silicon cantilevers and the reduced contact area, which is approximately 30 μm^2. To this end, a transfer method was developed whereby only a thin film of suspended PPC covered by the 2D material comes into contacts with the cantilevers. To enhance adhesion and reduce the native oxide before transfer, the substrate was subjected to plasma treatments based on O_2 and SF_6. To create the suspended PPC film, a hemisphere of PDMS is cut in four quarters, with a distance of a few hundred micrometers between each quarter. PPC is then deposited on a silicon substrate, which is subsequently removed with adhesive tape. The tape is perforated to create a suspended film of PPC, which is deposited on top of the previous PDMS structure. This process resulted in the structure shown in Figure S1-1.
During the pick-up step of the hBN flakes with these PPC structure, the temperature rises to 55 °C in order to improve adhesion. The second phase of the transfer process entails the meticulous placement of the flake onto the suspended cantilevers. To prevent excessive stress during this process, it is important to proceed slowly, with a duration of about two hours. Once a small contact between the cantilevers and the PPC is established, the temperature is gradually increased to 65°C. At this temperature, the PPC gradually softens and slowly contacts the surface. The temperature is then gradually elevated to 95°C to facilitate the gradual melting of the PPC. This process prevents any deformation. The PPC is then subjected to evaporation by placing the sample in an oven at 350°C for four hours under vacuum. With this process, the samples are free of contamination.

2. Typical samples

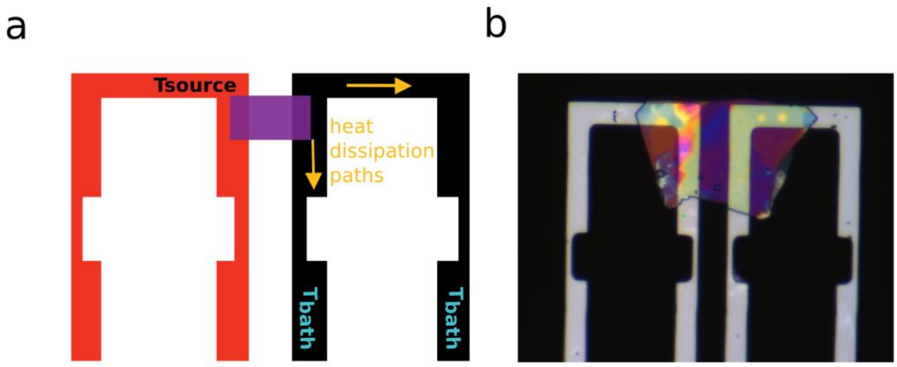

Figure S2-2: Schematic (a) and optical image (b) of the sample with a 2D material (purple), suspended between two microheaters in silicon. The red electrode is heat up to T_{source} by Joule heating. The two heat dissipation paths are marked in yellow.

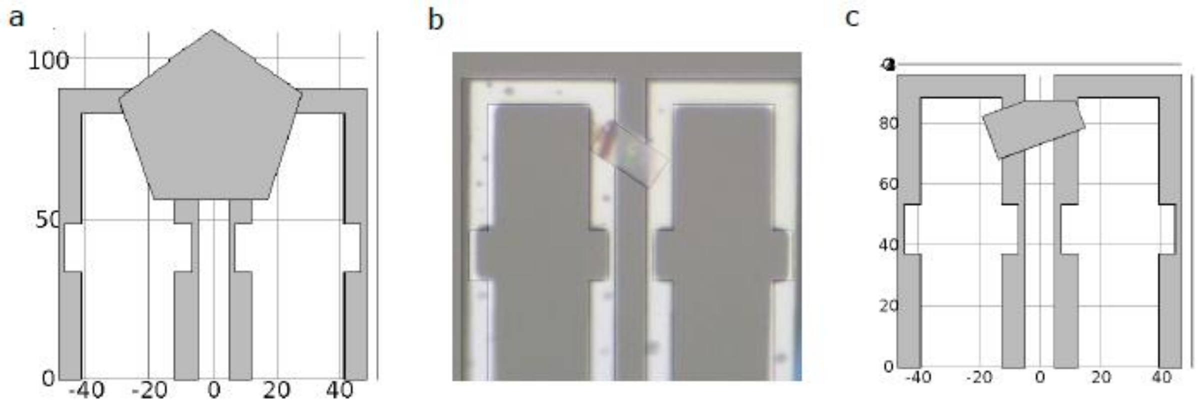

Figure 2-3: Geometric shapes used in the simulations. a) Geometry of HS1. b) Geometry of HS2 superposed with an optical image. c) Geometry of Gr1. Scales in micrometers.

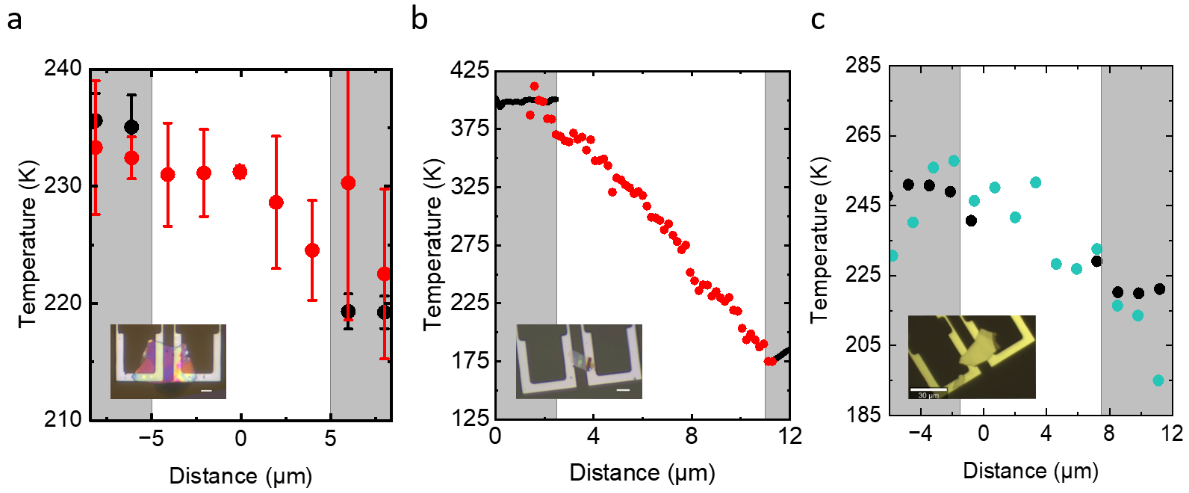

Figure 2-4: Temperature profiles along the heat propagation direction. Temperature of silicon (black), WSe_2 (red), graphite (blue) in a) HS1 with *P*=4.6 mW, T_{bath}=-109 °C. b) HS2 with *P*=6.2mW and T_{bath}=-116 °C. c) Gr1 with *P*=5.6 mW and T_{bath}=-127 °C. The grey areas correspond to the regions with cantilevers and the white area to the region with the suspended 2D material region. In insets are presented the optical images of the different samples.

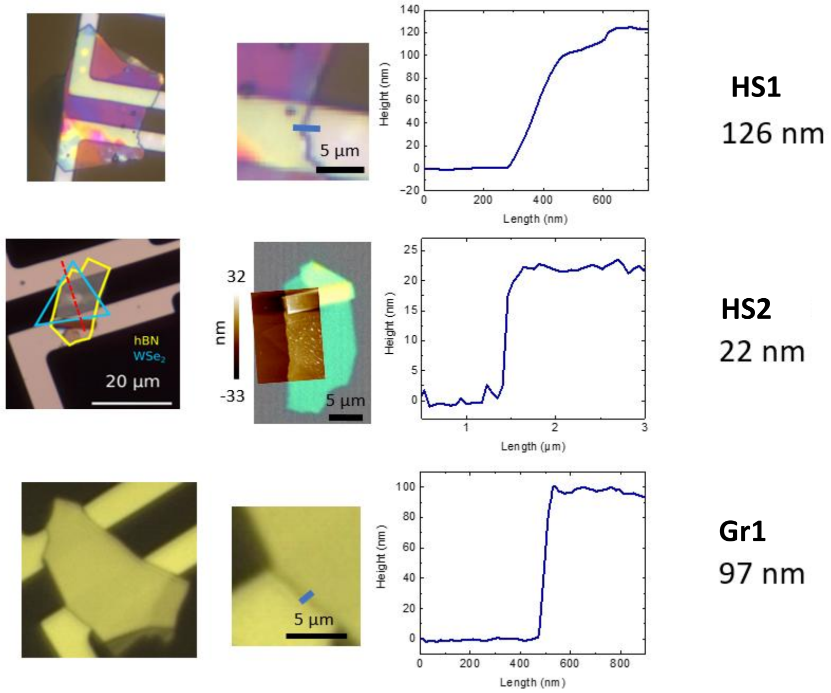

Figure S2-5: Atomic force microscopy of the different flakes. For Gr1 and HS1, the thicknesses were measured directly on the suspended membranes.

The electrical conductivity of silicon was determined by the geometry and the extracted electrical power. The temperature dependence of the electrical conductivity has been integrated. The electrical resistance of graphite was considerable, but to prevent any Joule heating of the graphite itself, the measurement was performed with the electrical contacts of the second microheater in floating mode, which was only thermally anchored to the substrate. It is noteworthy that the temperature difference between the two points on the Si cantilever was identical to the graphite temperature (same T_{hot} and T_{cold}). It indicates good thermal contacts and confirms the value of ΔT_{2D}.

3. Raman spectrum and temperature calibration

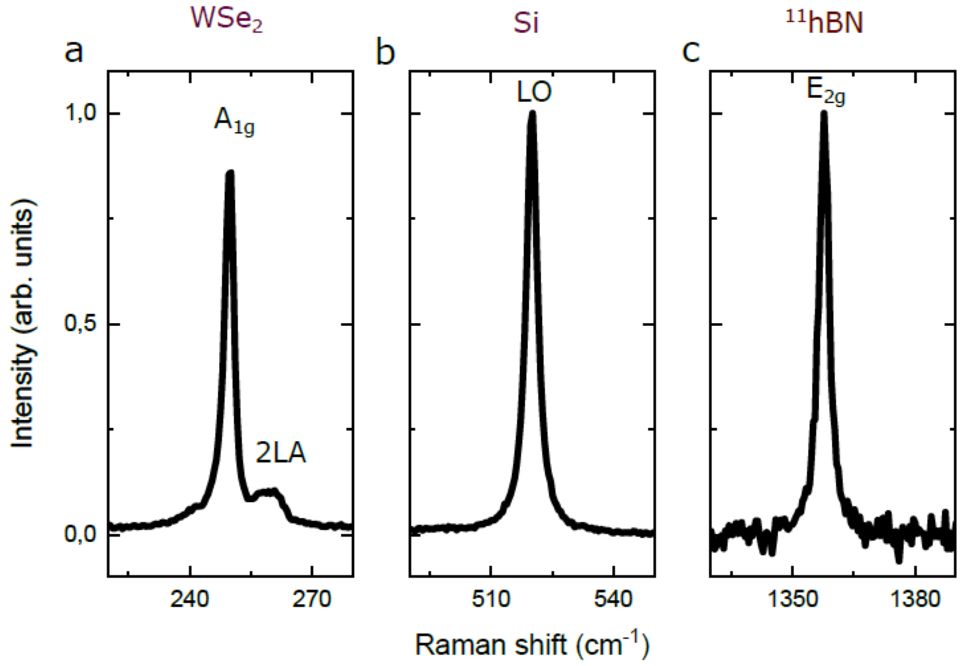

Figure S3-6: Stokes-Raman spectrum of a) WSe_2, b) silicon and c) hBN[11] at ambient temperature.

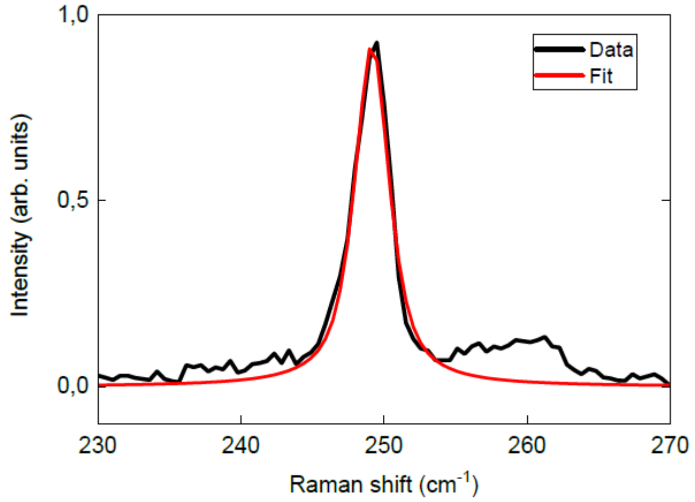

Figure S3-7: Raman spectrum of WSe_2 with fitting by a Pseudo-Voigt function

First-order thermal coefficient

At very low temperatures, the frequency ω of the characteristics vdW materials Raman peaks is almost constant [8–10], while at higher temperature a linear temperature dependence of ω is commonly used with $\omega(T) = \omega_0 + \chi T$ [11–14]. We measure the first-order thermal coefficient χ for different two-dimensional materials, such as TMD MoS_2 [11–14] or WSe_2 [15] but also for Graphene[16–18] and hBN [18–24] and for silicon [25–29].

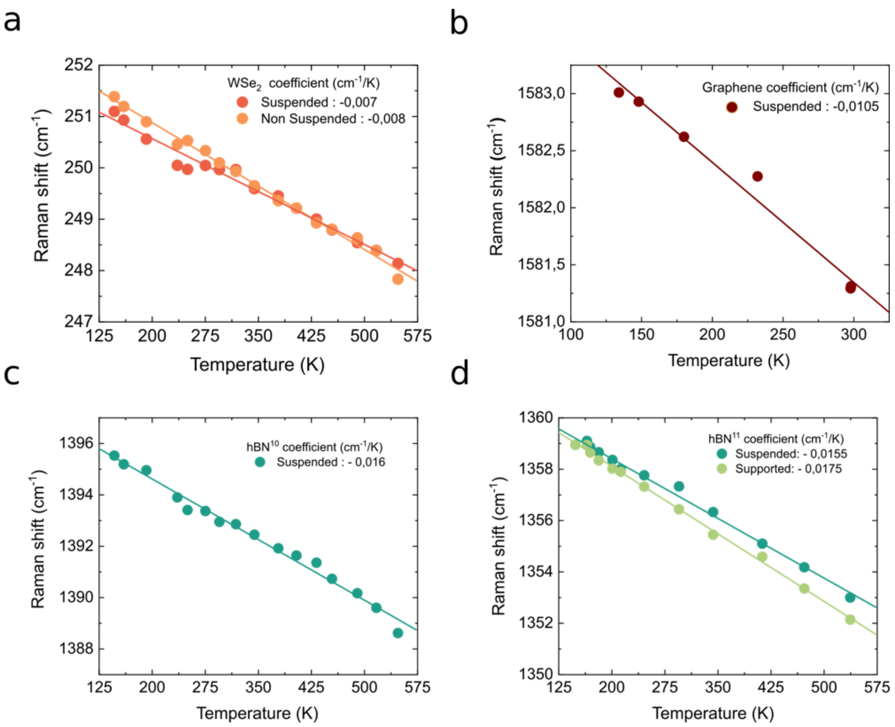

Figure S3-8: Raman peak position as a function of temperature for a) the A_{1g} WSe_2 peak, b) for the G peak of graphene, c) for the E_{2g} peak of hBN^{10}, d) for the E_{2g} peak of hBN^{11}.

Supplementary table 3a : First-order coefficient of Raman spectrum of hBN as extracted from the literature.

first-order Raman temperature coefficients χ $E_{2}g$ ($cm^{-1}.K^{-1}$)	Isotropy of h-BN	Layer number	Linear ?	Temperature range (K)	Suspended ?	Ref
Strain corrected<br>−0.0197 ± 0.0007	Natural	11	Yes	300-370	Suspended	[24]
Strain corrected<br>− 0.0170 ± 0.0007	h-BN 10	11	Yes	300-370	Suspended	
−0.0223 ± 0.0012,<br>−0.0214 ± 0.0010,<br>and −0.0215 ± 0.0007	Natural	1<br>2<br>3	Yes	300-400	Suspended	[23]
−0.0558 ± 0.0011,<br>−0.0480 ± 0.0022,<br>and − 0.0380 ± 0.0011	Natural	1<br>2<br>3	Yes	300-400	On substrate	
−0.0191 ± 0.0005	Natural	Bulk	Yes	300-400	Suspended	
−0.0341 ± 0.0012,<br>−0.0315 ± 0.0014,<br>and −0.0378 ± 0.0016	Natural	1<br>2<br>9	Yes	300-450	On substrate	[22]
-0.025	hBN 10, Natural, hBN 11	Single crystal	No	100-600	On substrate	[24]
−0.023	Natural	Bulk	Yes	300-680	On substrate	[20]
− 0.04123<br><br>− 0.02385	Natural	16.2 nm<br><br>36.2 nm	Second order poly.<br>-8.446 × 10−5<br>− 2.501 × 10−5<br>cm−1 °C^2	80-473	On substrate	[18]
-0.025	Natural	Bulk	With DFT	80-600	On substrate	[21]

Supplementary table 3b : First-order coefficient of Raman spectrum of Si as extracted from literature.

First-order Raman temperature coefficients χ $E_{2}g$ ($cm^{-1}.K^{-1}$)	Linear ?		Temperature range (K)	Article
-0.054	Yes		300-1200	[25]
-0.0216	No		10-800	[26]
From- 0.02 to -0.034 depending of the reference	Yes		240-400	[27]
−0.022	Yes		300-420	[28]

-0.047	Yes		180-800	[29]

Stokes and anti-Stokes calibration.

The temperature can be also determined by measuring the ratio between the intensity of the Stokes and anti-Stokes peaks. The probability of Stokes or anti-Stokes scattering depends on the density of active Raman phonons. The ratio between the two peaks dependents on the temperature [30–34]:

$$\frac{I_{AS}}{I_S} = \frac{(\nu_{laser} + \nu_{RS})^n}{(\nu_{laser} - \nu_{RS})^n} e^{\frac{-hc.\nu_{RS}}{k_B T}}$$

The temperature can therefore be writted as:

$$T = \frac{-hc.\nu_{RS}}{k_B T} \cdot \frac{1}{n\left(ln(\nu_{laser} + \nu_{RS}) - ln(\nu_{laser} - \nu_{RS}) + ln(I_S/I_{AS})\right)}$$

With I_{AS} the intensity of the (Anti-)Stokes Raman peak, ν_{laser} the frequency of the laser, ν_{RS} the frequency of the Raman peak and n a coefficient. In the literature, *n*=4 [30–33,35,36], *n*=3 [30,31,34,36,37] or even *n*=0 [36] have been reported. It is explained that under thermal equilibrium, n is dependent on the detection process [24,30,31].

Figure S3-9a shows the variation of the LO silicon Raman peak as a function of temperature. Figure S3-9 b illustrates the variation of the position of this silicon peak as a function of the temperature of the Linkam cell. Subsequently, a coefficient χ= -0.0187 cm^{-1}. K^{-1} is determined by linear extrapolation, based on the assumption that $\omega(T) = \omega_0 + \chi T$. In addition, the temperatures evaluated with the Stokes/Anti-Stokes intensity ratio according to the aforementioned formula with *n*= 4, 3 and 0 are plotted in red, orange and yellow, respectively. We note that there is a discrepancy of about 10 °C between the temperature of the Linkam cell and the Stokes/Anti-Stokes intensity ratio. As a consequence, we decide that the temperature of the Linkam cell should serve as an absolute reference between -200°C and 20°C. To compensate for this offset, a correction factor of 0.91, 0.89 and 0.83 was inserted into the previous formula for n = 4, 3 and 0 respectively. We note that the solutions for n=0 did not converge to the desired results. Calibration using the Stokes-Anti- Stokes measurement enables the calibration curve to be extended to temperatures higher than 20°C at the microheater, which is not possible with the Linkam cell temperature alone.

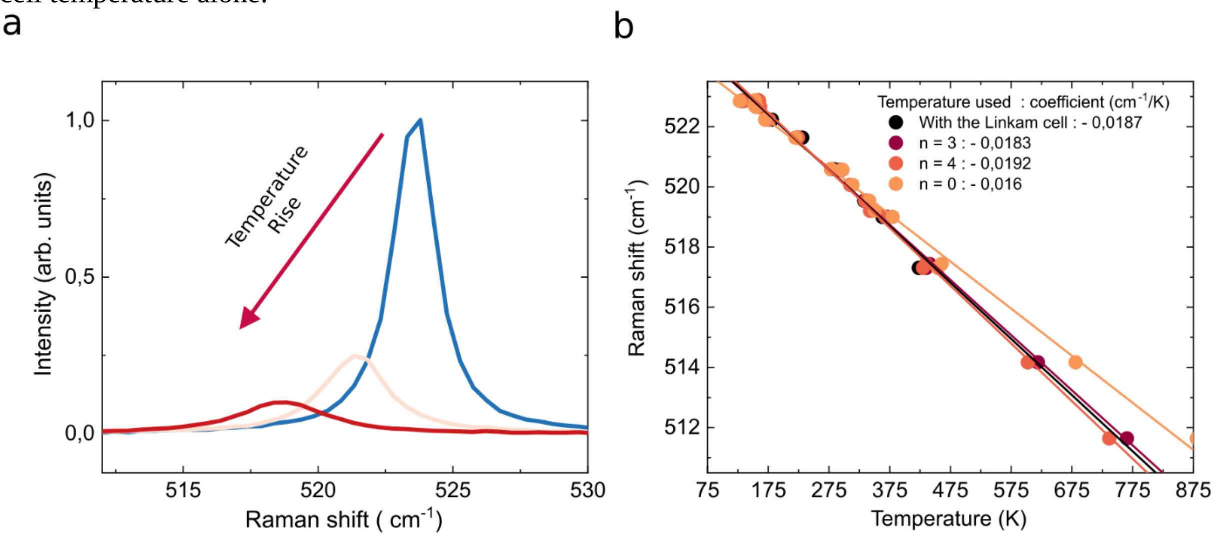

Figure S3-9: Temperature dependence of the Raman peak of silicon. a) Stokes Raman spectrum at three temperatures with the same laser power. b) Position of the Raman peak as a function of temperature with the different linear extrapolations.

4. Joule heating, laser heating and WSe_2 thermometry

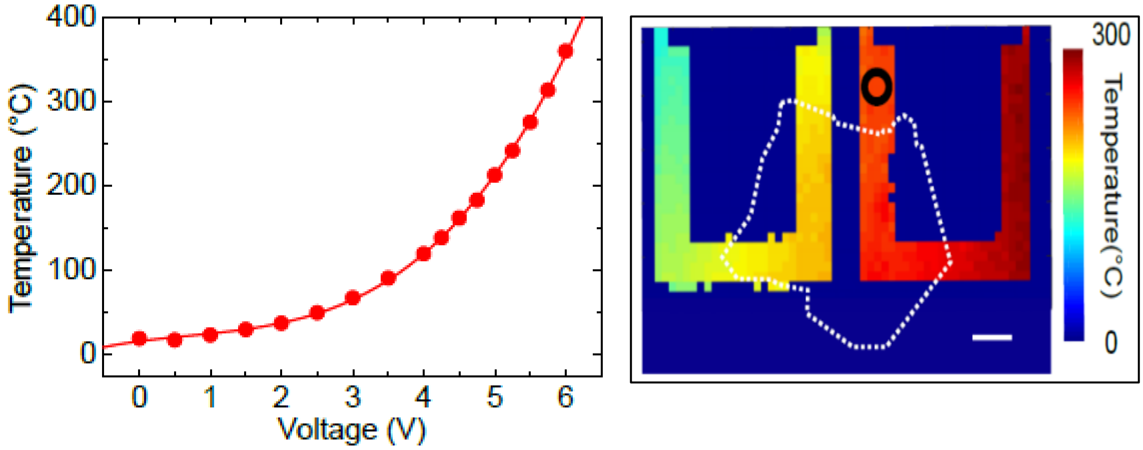

Figure S4-10: Joule heating in a silicon cantilever: a) Temperature of the silicon cantilever as a function of the applied voltage. b) Temperature map along the two cantilevers for HS1, extracted from the Si peak. The measurement was performed at T_{bath} =23°C.

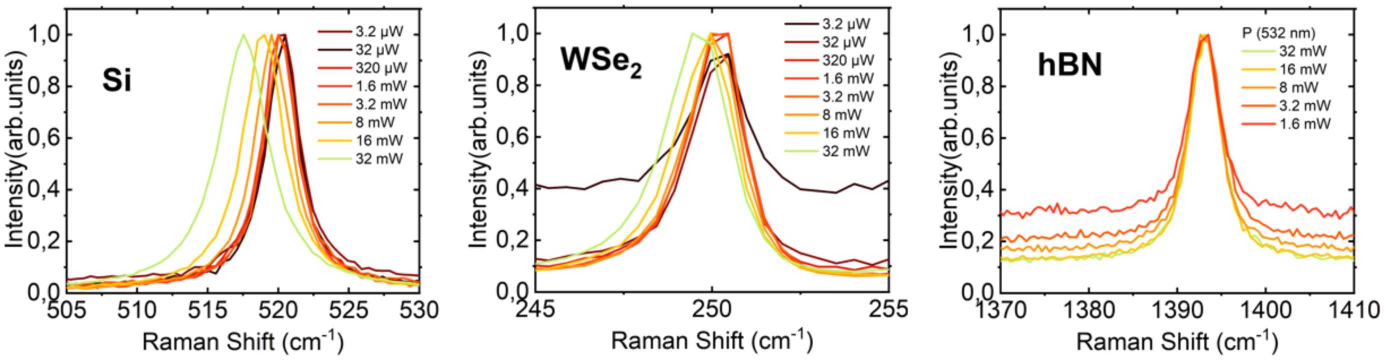

Figure S4-11: Raman signature of a suspended heterostructure with WSe_2 on top of hBN as a function of the applied optical power. (right) Raman signature of Si microheater as a function of the applied optical power. (middle) Raman signature of WSe_2 on top of the hBN as a function of the applied optical power P. (left) Raman signature of the hBN as a function of the applied optical power.
We do not observe a laser heating of hBN up to 3.6 mW.

a

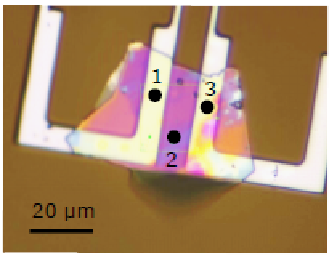

b

Points	hBN at 0 V (°C)	WSe_2 at 0 V (°C)	Si at 0 V (°C)	hBN at 6 V (°C)	WSe_2 at 6 V (°C)	Si at 6 V (°C)
1	- 104.7	- 104.8	- 120.7	- 37.5	- 41.5	- 51.9
2	- 82.3	- 93.6	/	- 41.1	- 40.9	/
3	- 94.7	- 94.0	- 120.8	- 50.1	- 44.1	- 70.2

Figure S4-12 : Temperature measurements at different points in HS1. a) Optical image of the sample HS1 with the three measurements positions in black. b) Table with the temperatures of the different materials at three positions and for V=0 V and V=6 V.

5. Absence of deformation and optical separation of strain from Raman measurements

Given the sensitivity of Raman spectroscopy to deformations, it is crucial to determine with precision whether mechanical deformations and forces are exerted on the cantilevers or on the 2D material. To achieve this, a variety of techniques were employed. The first step was to determine whether any optically discernible deformations occurred during the heating process, such as defocusing of the structure or the emergence of visible folds. The second approach involved using atomic force microscopy to assess the surface of the 2D material during the heating of the structure. This allows to verify the absence of any folds or bends at the nanometer scale. The third method is illustrated in Figure 1e and employs direct Raman spectroscopy to identify and separate the mechanical stress component from the temperature component on the Raman spectrum. This method enables to quantify the mechanical stress at 0.1%, which is negligible in the Raman signature of WSe_2 and can be separated in the data.

The fourth method refers to the measurements presented in Figure S2-4b. We note here that the temperature points for the silicon and two-dimensional materials are well aligned at the contacts level, indicating the absence of an relative strain. However, an asymmetry of strain between the silicon and the two-dimensional material is expected due to the geometric aspect ratios.

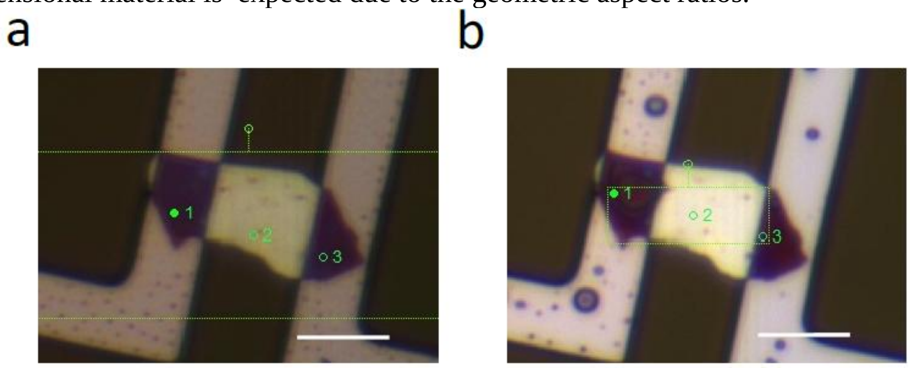

Figure 5-13: Optical image of the sample. (a) Without and (b) with electrical heating at V=5V at T_{bath}=-120 °C. Scale bar=10 µm. We do not see any strong deformation of the sample. This is confirmed for all samples.

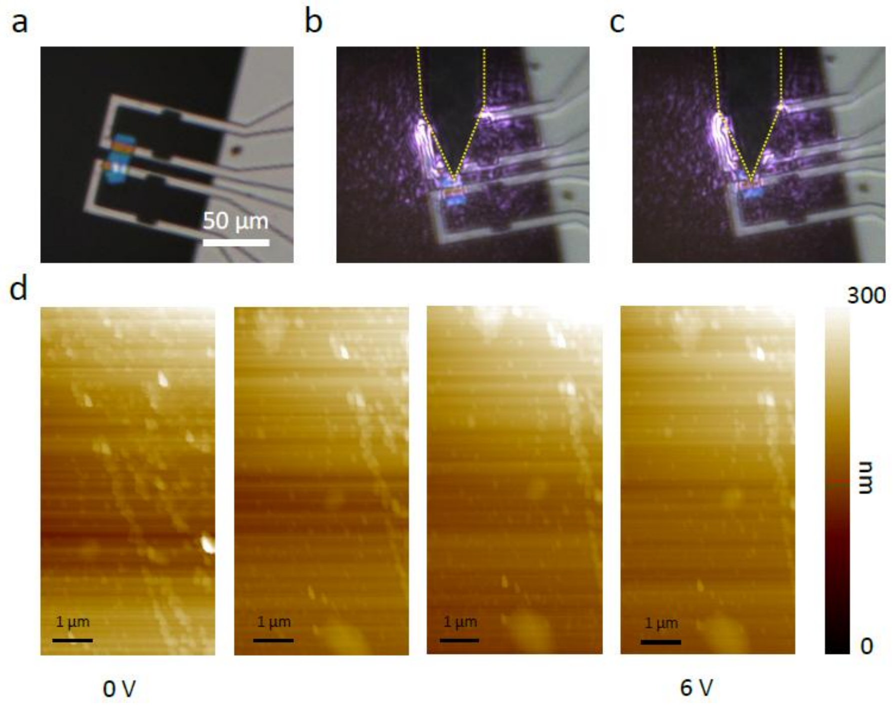

Figure 5-14: Atomic force microscopy of a suspended flake of hBN. a) Optical image of the sample, b) and c) Optical images of the sample during the atomic force microscopy measurements. d) topographies of a suspended part of the hBN with *V* between 0 and 6 V.

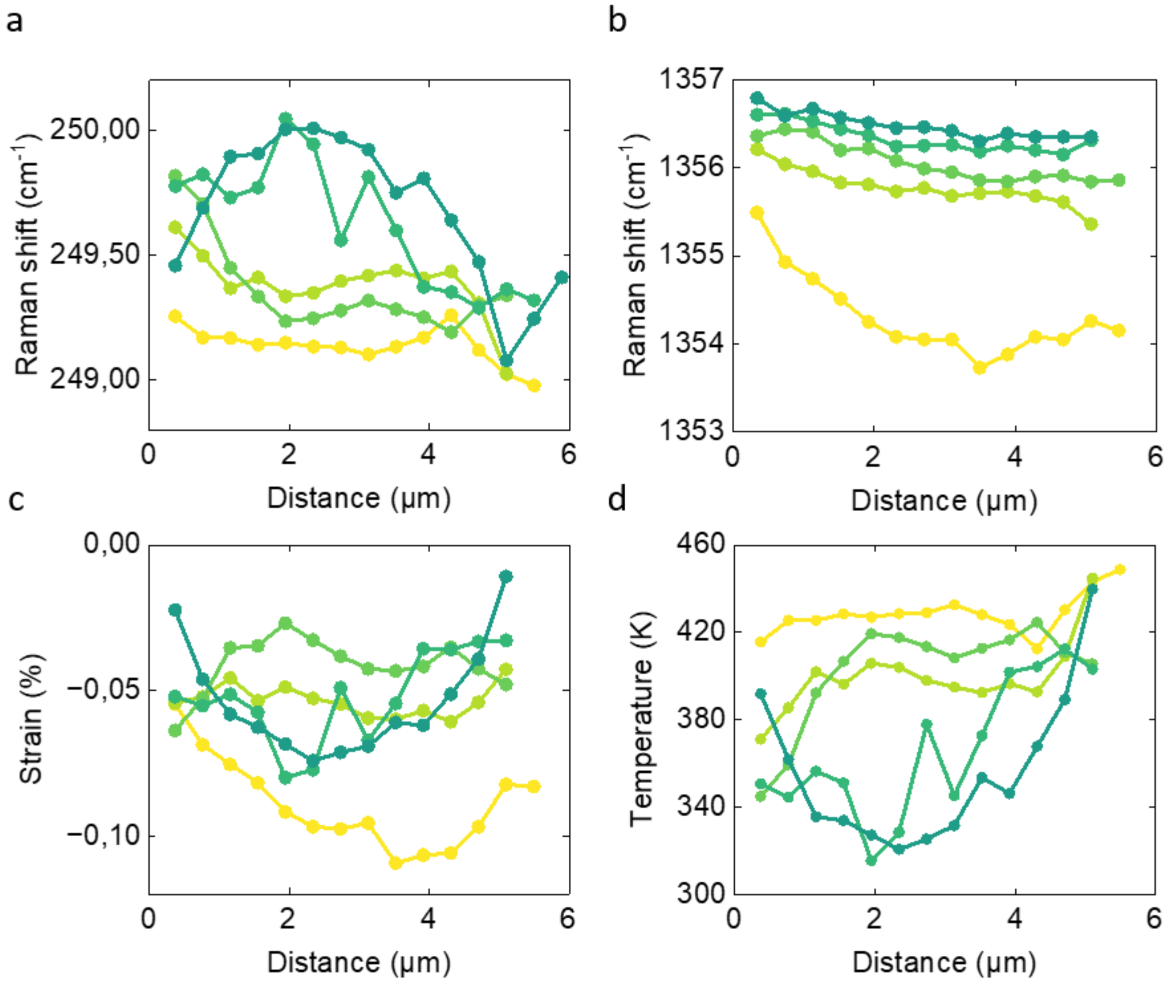

Figure S5-15: Optical separation of strain and temperature for sample HS3. a) Position of the E_{2g} peak of hBN at varying x-values (longitudinal direction) along the y-values (transverse direction). b) Position of the A_{1g} peak of WSe_2 at varying *x*-values along the *y*-values. c) Extracted values of strain (bidirectional) obtained for 2D heterostructures along the *y* direction at varying *x*-values. d) Same procedure for temperature.

It is possible to extract information from the Raman shift on both temperature and strain for the heterostructure composed of hBN and WSe_2. All data points presented in Figures S5-15 are included in Figure 1e in the main text. The data are aligned with the directions of the strain and temperature axis. They correspond to homogeneous deformations along the heterostructure and homogeneous heating or cooling of the heterostructure. This demonstrates that temperature and strain can be optically separated in our devices and that the WSe_2 flake is at the same temperature of the hBN flake. hBN is highly sensitive to strain [38], while the WSe_2 peak is mainly sensitive to temperature.

6. Temperature uncertainty and parasitic thermal effects

The determination of the temperature standard deviation is detailed here. We determine $\delta T_{Si} = 3°C$ for silicon, $\delta T_{WSe2} = 5°C - 8°C$ for WSe_2, and $\delta T_{Gr} = 3.5°C$ for graphene. Figure S6-16 shows the measured temperature for sample HS1 (a) and sample Gr1 (b) when no heating of the microheater is applied. From these measurements we can extract an uncertainty of 5°C for WSe_2, since data for each temperature value on the longitudinal *x*-axis is an average over 5 points on the *y*-axis (the standard deviation corresponds to the error bar in Figure 3a). It is confirmed that the temperature of the silicon holder was well defined at T_{bath}. This test also demonstrates that there is no parallel channel of thermal conduction between the two cantilevers, in addition to the conduction through the 2D material, neither through residual air conduction nor radiative thermal transport.

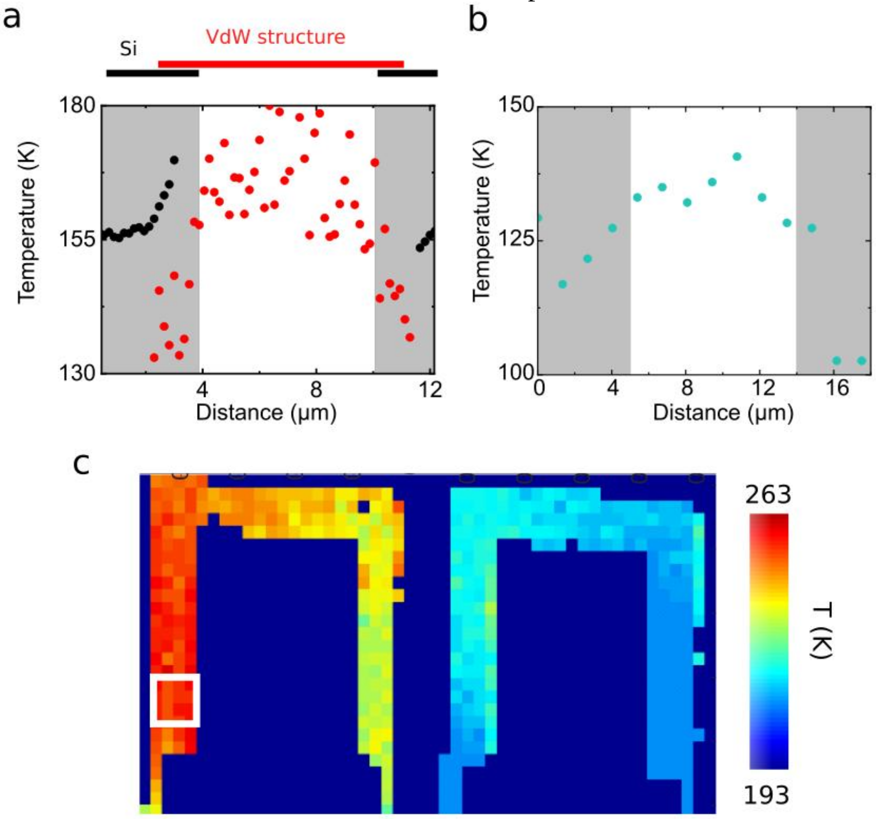

Figure S6-16: Determination of the standard deviation of the temperatures during the measurements. a) Temperature of WSe_2 (red) and Si (black) at *V*=0 V and T_{bath} =-118 °C along the sample HS1. The grey (white) areas correspond to the supported (suspended) sections. b) Temperature of the Graphite, illustrated in blue at *V*=0 V and T_{bath}=-127 °C in Gr1. c) Temperature map of the silicon cantilevers at *V*=6 V and T_{bath}=-109 °C for HS1. The white square indicates the area used to measure the standard deviation.

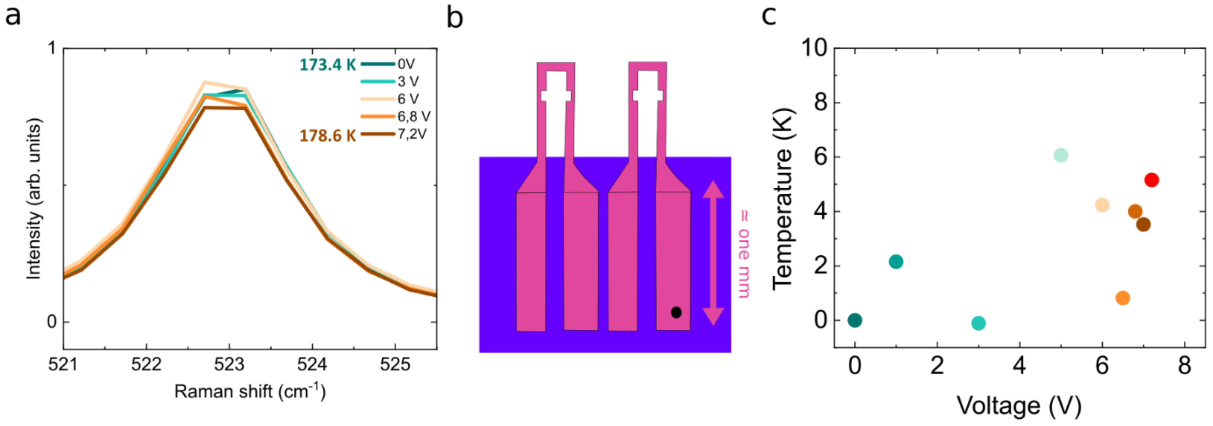

Figure S6-17: Thermal bath. a) Stokes Raman spectrum of silicon away from the hot zone for different voltages V. c) Temperatures variations evaluated from the position of the Si peak in a). The black dot in the sketch b highlights the measurement point.

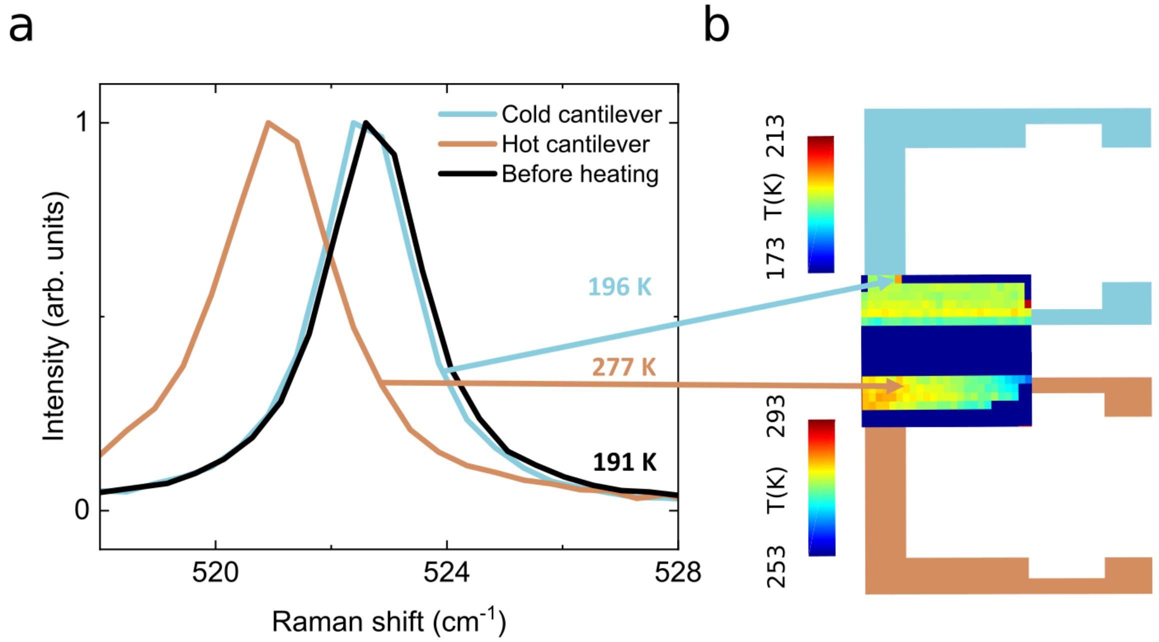

Figure S6-18: Parasitic thermal path with thermal radiation and thermal conduction through the residual air. a) Raman spectra of Silicon on the two cantilevers without suspended 2D material with and without Joule heating of the microheater with the corresponding temperatures extracted from the peak position. The different arrows indicate the measurement positions. b) Temperature map along the two cantilevers with Joule heating.

7. Temperature dependence of the electrical and thermal conductivity for HS1, HS2 and Gr1.

The majority of materials display a dependence on temperature of their thermal and electrical conductivities. Given the considerable temperature difference across silicon cantilevers (exceeding 100°C), it is imperative to consider this temperature dependence. The electrical conductivity of the microheaters, σ_{Si}, was determined independently using the initial electrical conditions (P = 4.6 mW, 6.2 mW and 5.6 mW for HS1, HS2 and Gr1, respectively) and a fitting parameter (α) from an external electrical measurement:

$$\sigma_{Si} = \alpha(5.10^{-11}T^2 - 10^{-8}T + 4.10^{-5})^{-1}$$

Figure S7-19 a shows the electrical conductivities of silicon for the three samples as a function of temperature. There is a slight discrepancy between the samples, which can be attributed to variations in the manufacturing process. However, they exhibit values that closely aligned with those observed in typical silicon samples. The electrical conductivities of heavily doped silicon, as in our cantilevers, are observed to be higher than those of intrinsic silicon, typically in the range of 1000 S/m. At temperatures below 400 K, the electrical conductivity exhibits a nearly constant behavior with respect to the temperature and decreases slight above this range. It can be concluded that the temperature dependence of σ_{Si} is not a significant factor in the system under consideration.

In contrast, the temperature dependence of the thermal conductivity is of significant importance. This temperature dependence is well documented in the literature and it decreases with increasing temperature. The curve of $k_{Si,ref}(T)$ was extracted from the reference [39] and adjusted according to the conductivity of the sample at 200 K as :

$$k_{Si}(T) = k_{Si,ref}(T)\frac{k_{Si}(200K)}{k_{Si,ref}(200K)}$$

With $k_{Si}(200\,K)$ which corresponds to the fitting parameter in Comsol. Figure S7-19 b) shows the thermal conductivity of HS1, HS2 and Gr1, which is between 124 and 156 W.m^{-1}.K^{-1} at room temperature. This finding aligns well with the values reported in the literature, which are typically in the range of 140 W.m^{-1}.K^{-1} [40,41].

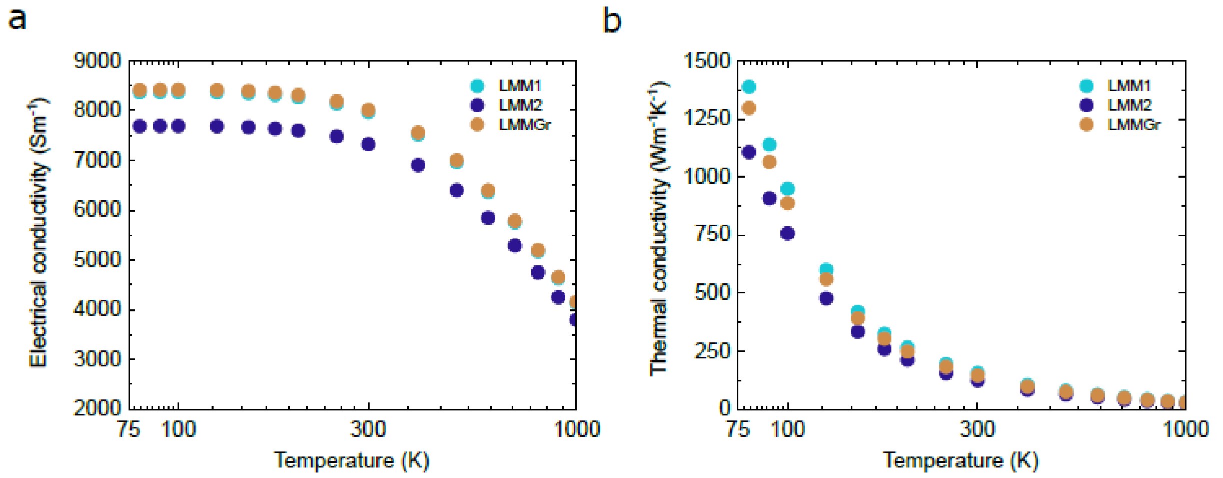

Figure S7-19: Temperature-dependent conductivities of silicon. a) Electrical conductivity of silicon as a function of temperature in the three samples. b) Typical thermal conductivity of silicon in the same samples.

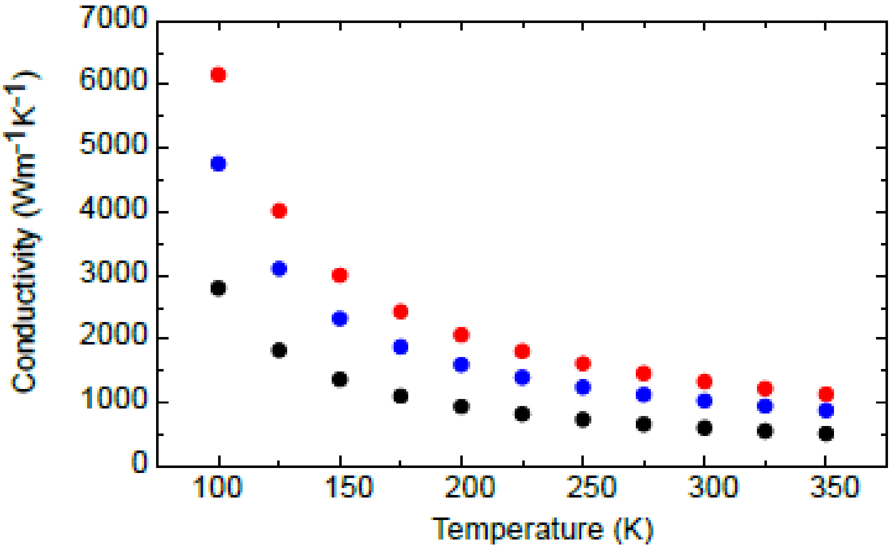

Figure S7-20: Temperature-dependent thermal conductivity of the vdW heterostructure used for the simulation in the sample HS2. In black is the curve from ref[42]. The red and blue dots delimit the maximum and minimum of thermal conductivities at each temperature obtained from the simulations for the sample HS2.

8. Simulations for GR1: 2-points configuration.

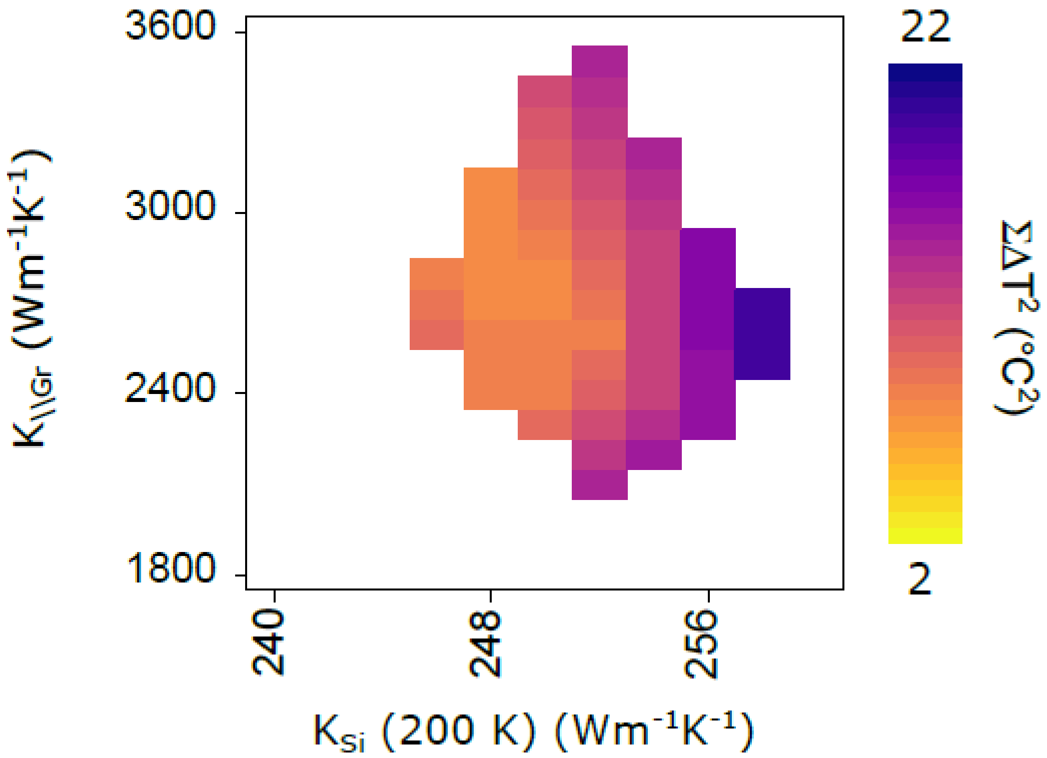

Figure S8-21: Phase space of the system with the best fitted solutions (colored region) as a function of k_{Si} and the in-plane k_{Gr} for the sample Gr1.

In this and the following section, we have defined the colored region in the different phase space graphs as the sum of the squares of the temperature differences for the two temperature points or more $\Sigma\Delta T^2 = \sum_{points}(T_{mesured} - T_{simulated})^2$. The white region describes the solution space in which at least one point of the simulated temperature $T_{simulated}$ has a difference to the measured temperature $T_{mesured}$ exceeding the standard deviation for at least one point ($|T_{mesured} - T_{simulated}| > \delta T$). The coloured region was defined as the region of the available solutions, and within this zone, the lowest value of $\Sigma\Delta T^2$ corresponds to the optimal solution.
This procedure enables the identification of the optimal solution for the given data set. To illustrate, if a single point diverges, ($|T_{mesured} - T_{simulated}| > \delta T$), this does not necessarily affect the value of $\Sigma\Delta T^2$. Our method addresses this issue.
The area of satisfactory solutions in phase space depends on the number of measurement points used. In the case of Gr1, a satisfactory solution can be obtained with only two points. We deduced from Figure S8-21 that the best solution is for k_{2D}=2800±700 W.m^{-1}.K^{-1}.

9. Simulations for HS2: temperature dependence of k_{2D}.

The temperature gradient measured along the HS2 at a voltage of 8 V is approximately 250 Kelvin. The thermal conductivity $k_{2D}(T)$ varies considerably within this range. This dependence can be derived directly from the temperature profile. Considering a bar of a material of length L, between two temperatures, T_{hot} and T_{cold}, it is possible to define the temperature profile in accordance with Fourier's law. To obtain the temperature along the x-longitudinal axis, it is possible to introduce a temperature dependence such as $k_{2D}(T) = cT^{\alpha}$. The temperature profile is then written as follows [43] :

$$T(x) = \left[\frac{x}{L}T_{cold}^{\alpha+1} + \left(1 - \frac{x}{L}\right)T_{hot}^{\alpha+1}\right]^{\frac{1}{\alpha+1}}$$

In Figure 4c, the temperature profile of the sample HS2 is quasi linear and corresponds to a coefficient α close to -1. It matches the expected behavior and experimental data obtained in reference [42].
A temperature dependence for $k_{2D}(T)$ was included in the COMSOL model. This model is based on the experimental values of $k_{2D,ref}(T)$ as reported in reference [42]. Thus, $k_{2D}(T)$ is defined as:

$$k_{2D}(T) = k_{2D,ref}(T)\frac{k_{2D}(190K)}{k_{2D,ref}(190K)}$$

Nevertheless, the inclusion of the temperature dependence in k_{2D} has a minimal impact on the conductivity value of the 2D material at room temperature, as shown in Figure S9-22 when comparing the thermal conductivity with temperature dependence at 300K and the thermal conductivity without temperature dependence We can compare at 300 K which corresponds to the mean of the vdW structure temperature.

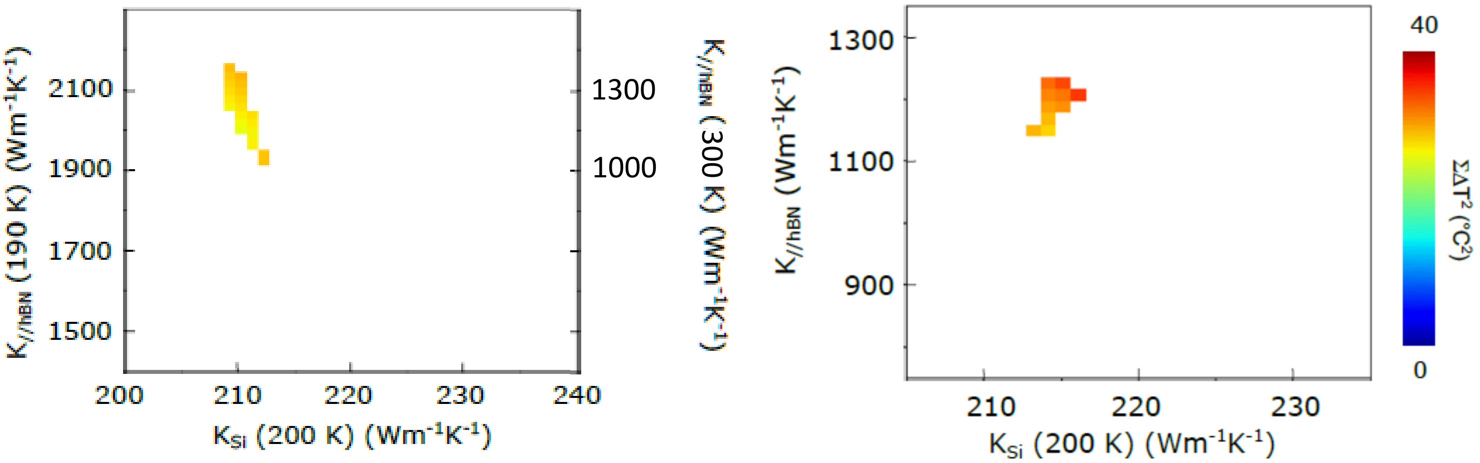

Figure S9-22: Simulations for the sample HS2 and phase space of the converging solutions a) with (left) and b) without (right) temperature dependence of k_{2D}.

10. Simulations for HS2: out-of-plane thermal conductivity

Figure S10-23 shows the optimization of the simulations with measurements of the sample HS2 for a range of out-of-plane thermal conductivities of the 2D material $k_{2D,\perp}$. It is not possible to achieve convergence for values of $k_{2D,\perp} = 2\,W.m^{-1}.K^{-1}$. The solutions are obtained for $k_{2D,\perp} = 5\,W.m^{-1}.K^{-1}$ and above. If $k_{2D,\perp}$ is greater than $5\,W.m^{-1}.K^{-1}$, the in-plane k_{2D} value is unaffected by the out-of-plane thermal conductivity of the material. This leads to the conclusion that the value of the out-of-plane thermal conductivity should be around $5\,W.m^{-1}.K^{-1}$. The table below shows the out-of-plane thermal conductivity values reported for hBN in the literature. The average value is close to 5 $W.m^{-1}.K^{-1}$, further validating our measurements.

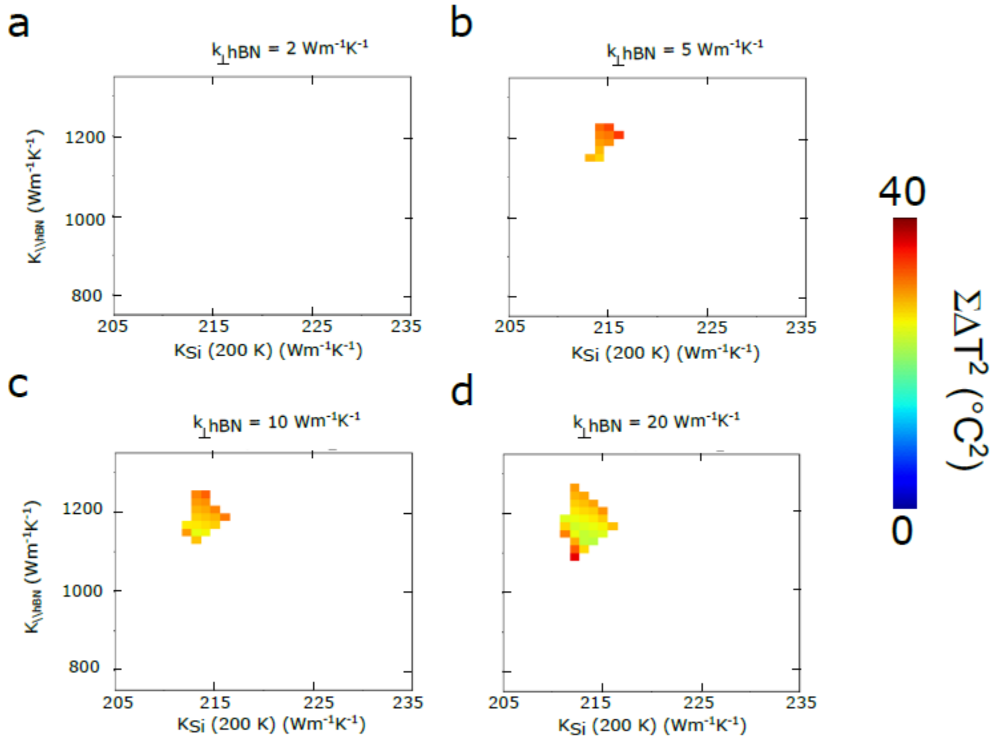

Figure S10-23: Simulations for the sample HS2. Phase space of the system with the fitted solutions (colored region) as a function of k_{Si} and the in-plane k_{hBN} for the sample HS2 at different out-of-plane thermal conductivities $k_{\perp hBN}$. a) $k_{\perp hBN}$=2 W.m^{-1}.K^{-1}. b) $k_{\perp hBN}$=5 W.m^{-1}.K^{-1}. c) $k_{\perp hBN}$=10 W.m^{-1}.K^{-1}. d) $k_{\perp hBN}$=20 W.m^{-1}.K^{-1}.

Supplementary table 10a : Out of plane thermal conductivity of others 2D materials from literature

Thermal conductivity (W/m/K)	Material	Temperature	Layers	Simu/Exp Suspendu e or not	Interface conductance (MW/m2/K)	Measure setup	Ref
4.4<br>3.8<br>2.8<br>2.5	MoS2<br>WS2<br>MoSe2<br>WSe2	Tamb	Bulk	Exp (Non)	/	Thermoreflectance (Change with the modulation frequency)	[44]
2<br>2	MoS2	Tamb	Bulk	Exp (Non)	26 MoS2/Al<br>20	TR-MOKE<br>Thermoreflectance	[45]
	MoS2<br>MoSe2	Tamb	1L<br>1L	Exp (Non)	0.44 MoS2/Au<br>0.09 MoSe2/SiO2		[46]
3.5	MoS2	Tamb	Bulk	Simu			[47]
1.5	WSe2	Tamb	Bulk			Thermobridge	[48]

Supplementary table 10b : Out-of-plane thermal conductivity of hBN from literature

Thermal conductivity (W/m/K)	Isotope/Non-isotope	Temperature	Layers	Simu/Exp	Measure setup	Ref
3,5<br>4,5<br>3,3	10<br>11	Tamb<br>Tamb<br>Tamb	Bulk	Exp	Thermoreflectance	[42]
8.1<br>0.2		Tamb	585 nm<br>7 nm	Exp	3ω style	[49]
2		Tamb	Bulk	Exp		[50]
5.2		Tamb	Bulk	Exp	Thermoreflectance	[51]
5,5		Tamb	Bulk	Simu	Thermoreflectance	[52]

11. Simulations for HS1: thermal contact simulation

Figure S11.24 shows the convergence of the fitting solutions at different k_{2D} and k_{Si}, the thermal conductivities of the two materials for the sample HS1, with three different values of thermal conductivity at the interface $k_{interface}$. The interface is defined and simulated according to the specifications shown in Figure S11-24. Figure S11-24 b illustrates a perfect circle within the phase space of the solutions, while Figures S11-24 a and S11-24 c show imperfect circles. In cases a and c, the temperature is closely aligned with the experimental values for one material but divergent for the other one. To interpret the results, it is useful to make an analogy with optics and the astigmatism of an optical beam through a lens. A light beam without astigmatism generates a perfect circle, while a beam with astigmatism produces misshapen circles. Astigmatism can be attributed to the presence of an additional angle (or additional parameter) between the plane and the direction of the beam. The $k_{interface}$ appears to behave in a manner analogous to this third parameter. Based on these considerations, we consider the scenario where $k_{interface}$= 1.4 W.m^{-1}.K^{-1} to be the most accurate.

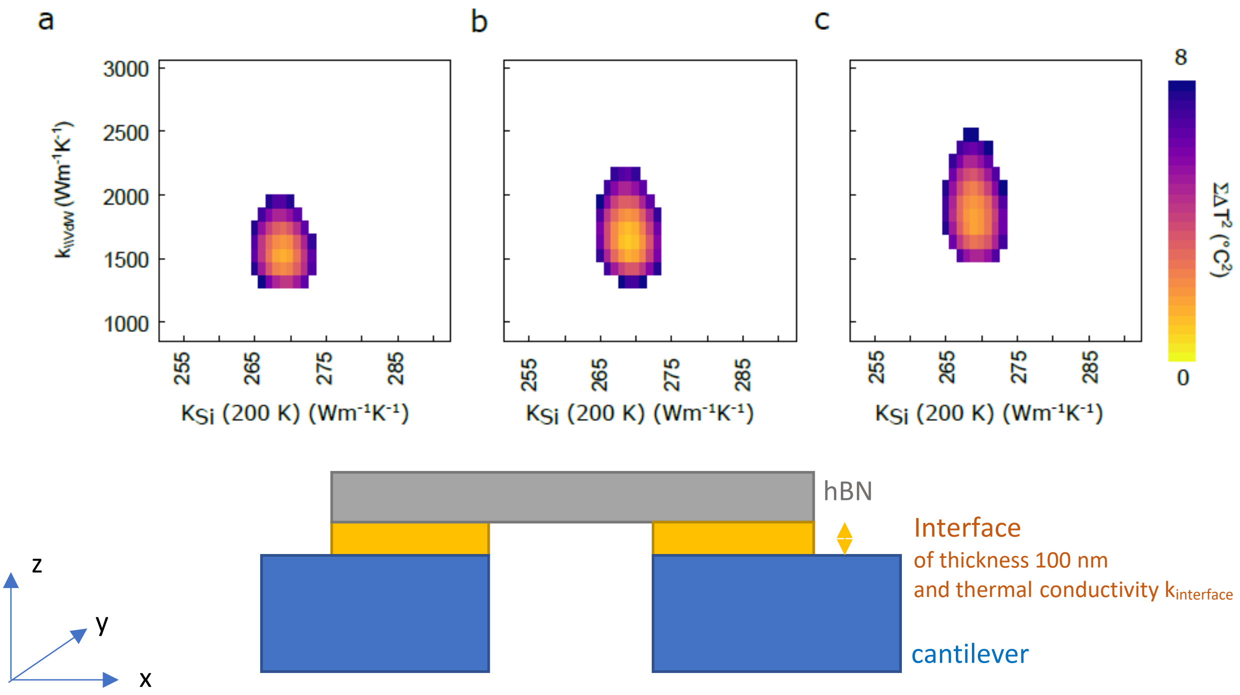

Figure S11-24: Simulations for the sample HS1. Phase space of the system with the correct solutions (colored region) as a function of k_{Si} and in-plane k_{hBN} for the sample HS1 at different contact thermal conductivity $k_{interface}$. a) $k_{interface}$=1 W.m^{-1}.K^{-1}. b) $k_{interface}$=1.4 W.m^{-1}.K^{-1}. c) $k_{interface}$=2 W.m^{-1}.K^{-1}.

$k_{interface}$ is the conductivity of an additional material, sandwiched between the hBN and the cantilever in order to simulate the thermal interface contact.
The electrical conductivity is equal to the 2D material's electrical conductivity (insulating for hBN).
For the interface, the thermal conductivity along x and y is 0 W.m^{-1}.K^{-1} to eliminate parasitic thermal transport and the vertical thermal conductivity is $k_{interface}$.

12. Thermal conductivity of hBN and graphene in the literature.

In recent years, several techniques have been employed to measure the thermal conductivity of van der Waals (VdW) materials. Some techniques require the use of supported materials, while others require the use of suspended samples, which ultimately leads to different thermal conductivities. Furthermore, the influence of temperature, sample thickness and the isotopic nature of the materials on thermal conductivity must not be ignored. The exceptionally high value of the thermal conductivity observed in one of our samples (k_{2D} =1650 W.m^{-1}.K^{-1} in HS1) is explained here. We conclude that our configuration for HS1 is favorable for a high value of k_{2D}.

Supported/suspended: As shown in Figure 2c, supported samples have overall lower thermal conductivities than suspended samples. This is obvious for graphene, with a ratio of 3 between the supported and suspended cases. For hBN, we extract a ratio of 1.7 with an analogous comparison. Similar comparisons have been made in previous studies collected on different 2D materials, in Figure S12-25, with an overall ratio of about 2.7 between suspended and supported samples. In addition, we compared the thermal conductivity of exfoliated isotopically pure hBN of a supported sample measured by modulated thermoreflectance (MTR) with the ones measured on the suspended samples. The exfoliated flake on the support measured by MTR has a thermal conductivity of 470 W.m^{-1}.K^{-1} at room temperature, i.e. there is a ratio of 3 between this value and the value obtained with the suspended flakes measured with the microheater configuration, which is reasonably expected according to the literature.

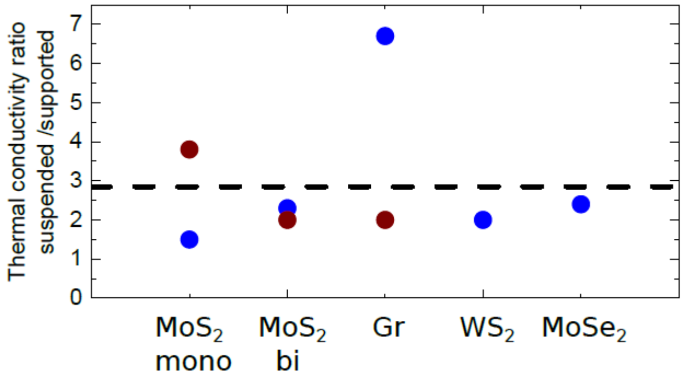

Figure S12-25: Compilation of the ratio of the thermal conductivity observed on the suspended and supported samples. Each of these ratios was carried out in the same article with very similar samples each time. The references are provided in the table below. In blue are the experimental data, and in brown the theoretical work.

Supplementary table 12a : Suspended/supported

Thermal conductivity (suspended/supported) in W.m^{-1}K^{-1}	Ratio	Temperature	Layers and material	Simu/exp	Ref
117/31<br>95/47	3.8<br>2.0	Tamb	1L Mos2<br>2L MoS2	Simu	[53]
2500/370	6.7	Tamb	1L Graphene CVD	Raman	[54]
1200/600	2.0	Tamb	1L Graphene	Simu	[55]
63/32	2.0	Tamb	1L Ws2	PL	[56]
84/55<br>59/24<br>77/34<br>42/17	1.5<br>2.4<br>2.3<br>2.4	Tamb	1L MoS2<br>1L MoSe2<br>2L Mos2<br>2L Mose2	Raman	[46]

Isotopic purity: The thermal conductivities of isotopic two-dimensional materials are typically higher than those of their natural counterparts. In reference [57], the authors performed thermal conductivity measurements at different isotopic concentrations in Graphene and discovered that pure isotopes exhibited thermal conductivity values twice those of natural carbon-based materials. Figure S12-26

shows that isotopically pure hBN exhibits higher thermal conductivity than natural hBN. We compare the thermal conductivity of exfoliated commercial hBN and exfoliated isotopically pure hBN with modulated thermoreflectance on supported samples. The thermal conductivity measured at room temperature was approximately 250 and 284 W.m^{-1}.K^{-1} for natural hBN and 470 W.m^{-1}.K^{-1} for isotopic hBN, respectively. The complete experimental description for natural hBN is provided in reference [58]. The thermal conductivity is observed to double from the utilization of an isotopic material.

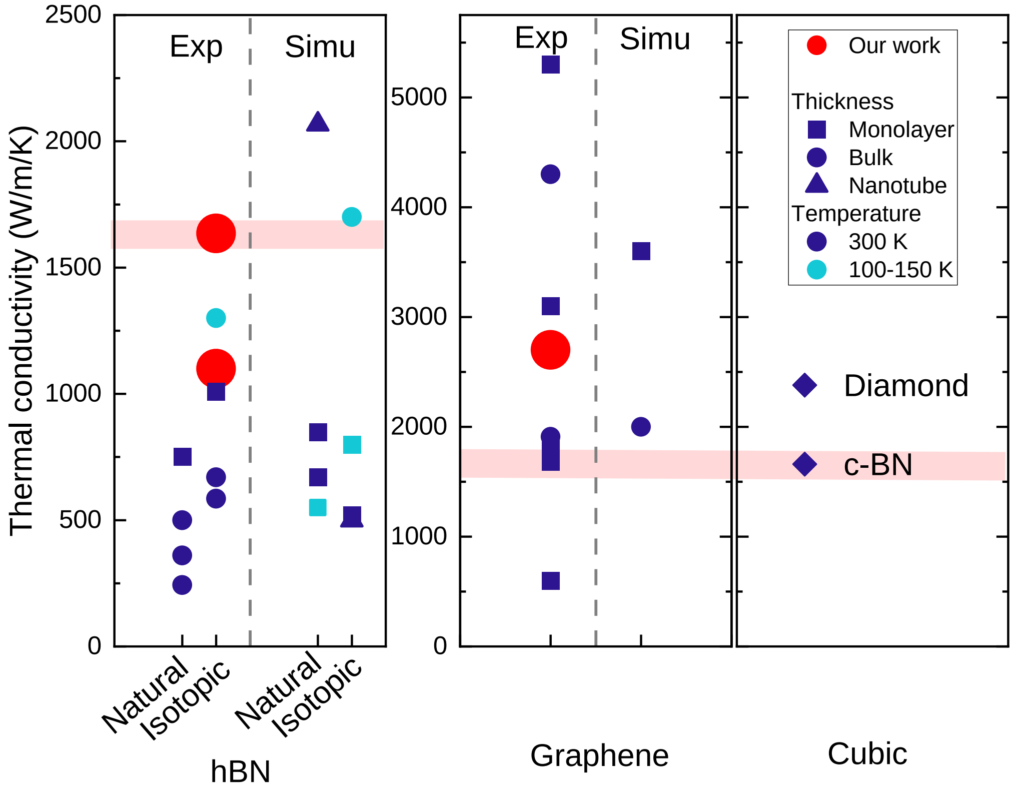

Figure S12-26: Comparative analysis of hBN and graphene/graphite thermal conductivities from the existing literature with the thermal conductivities evaluated in this study. We separate experimental and simulated measurements, isotopic and natural materials, ambient and low-temperature measurements, as well as the different thicknesses of the 2D materials. This graphic encompasses a multitude of thermal conductivity measurement techniques. All data and references are detailed in the table below.

Supplementary table 12b : In-plane thermal conductivity of hBN- graphene/graphite and different TMDs from literature

Material	Thermal conductivity (W/m/K)	Suspended/ supported	Isotope	Temperature	Thickness	Typical size	Setup	Ref.
hBN	585<br>550<br>408<br>1300	Sup	10<br>11<br><br>10/11	300 K<br>300 K<br>300 K<br>Tmax	Bulk	mms	Thermoreflectance	42
hBN	450	Sup		300 K	Bulk		Method 3ω	59
hBN	315	Sup		300 K	Bulk		Thermoreflectance	51
hBN	242	Sup		300 K	4L		Pump probe	60
hBN	1009<br>958	Sus	11<br>10	300 K	1L<br>1L	3.8 µm	Optothermal Raman	61
hBN	751	Sus		300 K	1L	3.8 µm	Optothermal Raman	23
hBN	670	Sus	10	300 K	11L	5 µm	Optothermal Raman	24
hBN	484	Sus		300 K	2L	3 µm	Thermobridge	62
hBN	360	Sus		300 K	11L	7.5/6.5 µm	Thermobridge	63
hBN	350	Sus	11	300 K	MWNT	6 µm	Thermobridge	64
hBN	243	Sus		300 K	9L	7µm	Raman	22
hBN	2067			300 K	BNNR		Simu	65
hBN	1650<br>900		10	Tmax	SWNT		Simu	66
hBN	1160				Bulk		Simu	67
hBN	670<br>730		11<br>11		1L		Simu	68
hBN	1700<br>600			125 K<br>300 K			Simu	42
hBN	550<br>800<br>520		 11<br>11	Tmax<br>Tmax<br>300 K	1L<br>1L<br>1L		Simu	69
hBN	550				Bulk		Simu	70
hBN	50–500		11	300 K	BNNT		Simu	71
Material	Thermal conductivity (W/m/K)	Suspended/ supported	Isotope	Temperature	Thickness	Typical size	Setup	Ref.
Gr	1910*	Sup		300 K	Bulk		Simu	72*
Gr	1900	Sup		300 K	Bulk		Thermoreflectance	51
Gr	1680	Sup		300 K	1L			73
Gr	~1000<br>~500	Sup		300 K	36L<br>1L		bridge	74
Gr	160<br>1000	Sup		300 K	1L<br>36L		Electric heat spreader	75
Gr	840<br>1100	Sup<br>Sus		300 K	1L		Raman	76
Gr	695	Sup		300 K	1L		FTDR	77
Gr	636	Sup		300 K	1L		Pump probe	60
Gr	600	Sup		300 K	1L		Microbridge	78
Gr	370<br>2500	Sup<br>Sus		350 K	1L		Raman	54
Gr	365	Sup		300 K	1L		Raman	79
Gr	308	Sup		300 K	5L		Raman	80
Gr	4840-5300	Sus		300 K	1L	3 µm	Raman	16
Gr	4300	Sus		300K	8.5 µm	mm	Thermobridge	81
Gr	4000	Sus	13/12	320 K	1L		Raman	57
Gr	2700	Sus		300 K	1L		Raman	82
Gr	2600-3100	Sus		300 K	1L	µm	Raman	83
Gr	370<br>2500	Sup<br>Sus		350 K	1L	3.8 µm	Raman	54
Gr	1875	Sus		350 K	1L		Raman	84
Gr	1800	Sus		300 K	1L	2.6 µm<br>6.6 µm	Raman	85
Gr	1800	Sus		300 K	1L		Raman	86
Gr	840<br>1100	Sup<br>Sus		300 K	1L		Raman	76
Gr	600	Sus		300 K	1L		Raman	87

Gr	3600<br>2200<br>2000	Simu		300 K	1L<br>2L<br>Bulk			88
Material	Thermal conductivity (W/m/K)	Suspended/ supported	Isotope	Temperature	Thickness	Typical size	Set-up	Ref.
$MoSe_2$	20	Sus		300 K	2L	7.5µm	Raman	89
MoS_2<br>WS_2<br>$MoSe_2$<br>WSe_2	82<br>120<br>35<br>42	Sup		300 K	Bulk		Thermoreflectance	44
MoS_2	80	Sup		300 K	Bulk		Thermoreflectance	51
MoS_2	62<br>15	Sup		Tmax<br>300 K	Bulk		Thermobridge	90
MoS_2	85-100	Sup		300 K	Bulk		TR magneto-optic Kerr effect	45
MoS_2	34.5	Sup		300 K	1L		Raman	91
MoS_2<br>$MoSe_2$<br>MoS_2<br>$MoSe_2$	84<br>59<br>55<br>24	Sus<br>Sus<br>Sup<br>Sup		300 K<br>300 K<br>300 K<br>300 K	1L<br>1L<br>1L<br>1L		Optothermal Raman	46
MoS_2	44-50	Sus		300 K	4L		Thermobrigde	92
WSe_2	124	Sup		300 K	Bulk		Thermobridge	48
WS_2	63	Sus		300 K	1L		PL	56
WS_2	32	Sup		300 K	1L		PL	56
WS_2	120	Sus		300 K	1L		Raman	93
MoS_2	90	Sus		300 K	1L		Raman	93
WSe_2	75	Sus		300 K	1L		Raman	93
MoS_2	68	Sup		300 K	5L		Raman	80
MoS_2	40	Sus		300 K	12L		Raman	94
$MoSe_2$	105	Sus		300 K	1L		Optomechanic	95
MoS_2	73	Simu		400 K	Bulk			47

Supplementary table 12c : Thermal conductivity of cubic structure from literature

Article	Thermal conductivity (W/m/K)	Isotope/Non-iso	Temperature (K)	Layers	Simu/Exp
96	1660<br>1650	11<br>10	Tamb<br>Tamb	Bulk c-BN	Exp
97	2380		Tamb	Diamond	Exp

Thickness of the material: As shown in Figure S12-25, the overall thermal conductivities (represented by the dots) are typically higher than those observed for monolayers (represented by the squares) in Graphene/graphite. In light of the findings from the study by Balandin et al.[16], the scientific community has anticipated that monolayer materials will exhibit a thermal conductivity that exceeds that of the bulk material. Within the community, this statement, the distinction between the two types of materials, has become less clear-cut, and the highest thermal conductivity is now observed in solid materials [81]. This may be attributed to the inherent challenges associated with measuring the thermal conductivity of monolayers. Recently, a reverse trend has been observed for TMDs[89]. The thermal conductivity of our thick samples' is higher than that of previously measured monolayers. Moreover, the results of our experiments indicate that the thinnest sample exhibits a lower conductivity than the thickest sample. The data suggest that the thermal conductivity is higher for thicker hBN. However, further investigation is required to confirm this hypothesis.

Measurement temperature: The maximum thermal conductivity in VdW materials is measured around 100 K [81,98]. In HS1, thermal conductivity is measured at around -50 °C and for Gr1, it is around -40 °C. These temperatures are slightly lower than the ambient temperature. Thermal conductivity in HS2 is measured between 120 and -100°C. Thermal conductivity is higher for measurements at low temperatures. This is why the measured values are expected to be slightly higher than the ambient temperature references, but the effect should be negligible.

13. Thermoreflectance on the supported isotopic hBN

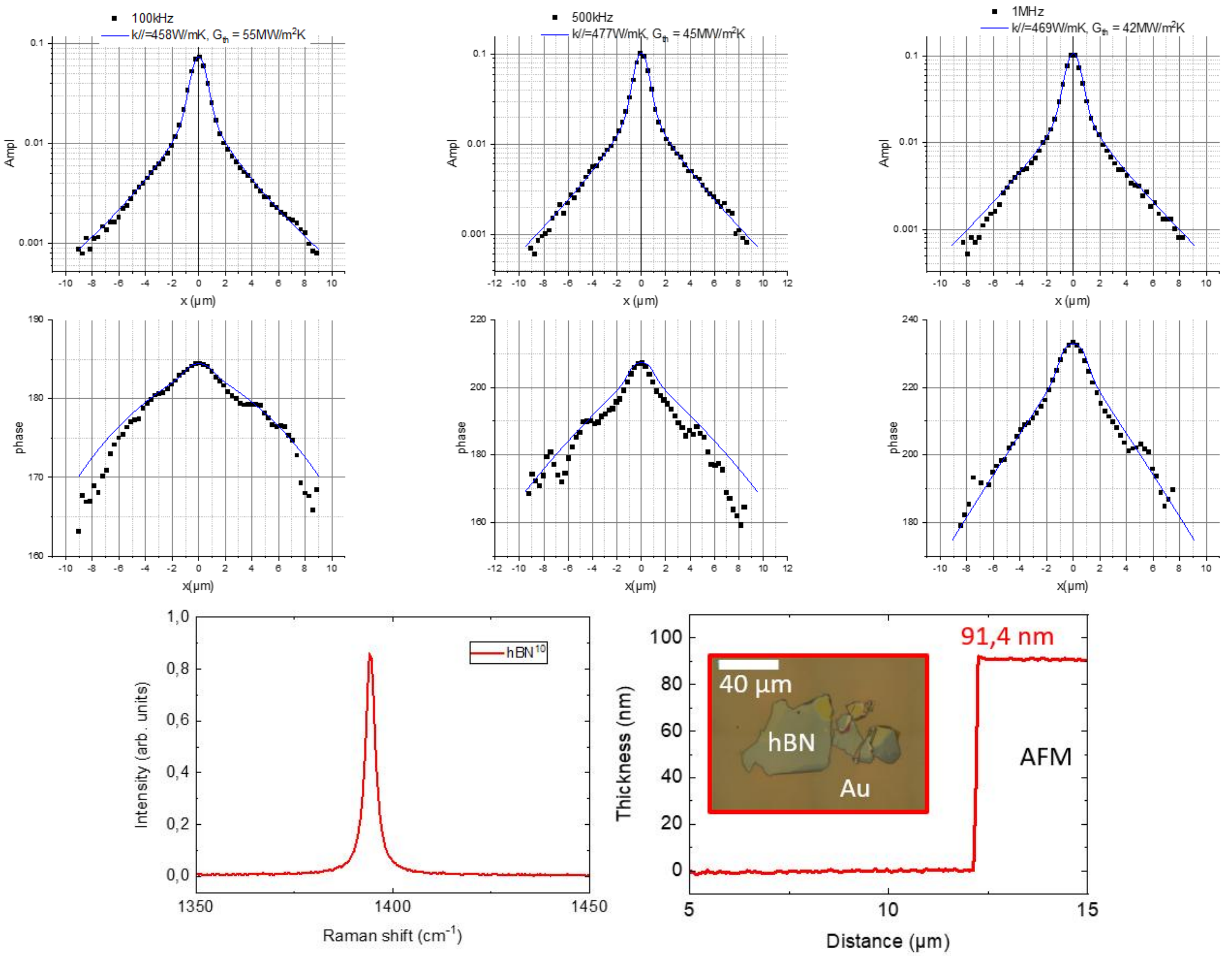

Figure S13-27 Thermoreflectance measurement reported in a thick flake of isotopic hBN deposited on a gold layer.

A study was carried out to measure and simulate the modulated thermoreflectance (MTR) of an analogous flake of hBN isotope. Details of the methodology are described in the reference [58]. We use a pump laser (green laser λ=532 nm at P~1 mW) modulated at a frequency in the range 100 Hz-1 MHz, and measure the variation of the reflectivity of a probe laser (bleu laser λ= 483nm at P~150 μW) as a function of their relative distance. The probe laser exhibited a response profile, as shown in the different graphics of Figure S13-27, related to the temperature gradient induces by the pump beam. We perform measurements at different frequencies of the laser modulation allowing to change the thermal diffusion length into the hBN. The amplitude and phase of the signal are plotted for each frequency measurement. Data are analyzed by solving a three-dimensional theoretical model of classical heat transport describing the temperature distribution in multilayered samples, heated by an intensity-modulated Gaussian laser beam, including both lateral and vertical heat diffusion [99]. The measurements fitting is conducted by including a surface thermal conductance G_{th} on the 91 nm layer of hBN isotope. The out-of-plane value

of k_{hBN} was set to 5 W.m^{-1}.K^{-1}, and G_{th} and $k_{hBN//}$ were used as fit parameters. The resulting values for G_{th} and $k_{hBN//}$ were approximately 47 MW.m^{-2}.K^{-1} and 470 W.m^{-1}.K^{-1}, respectively. Analogous MTR measurements of non-isotopic hBN flakes of slightly lower thicknesses (30-40 nm) have been already reported in literature [100]resulting in lower $k_{hBN//}$ values, of the order of 250 W.m^{-1}.K^{-1}, almost twice lower than what extracted for the isotopic hBN flake.

14. Example of spectra during the measurements (Figure 4a)

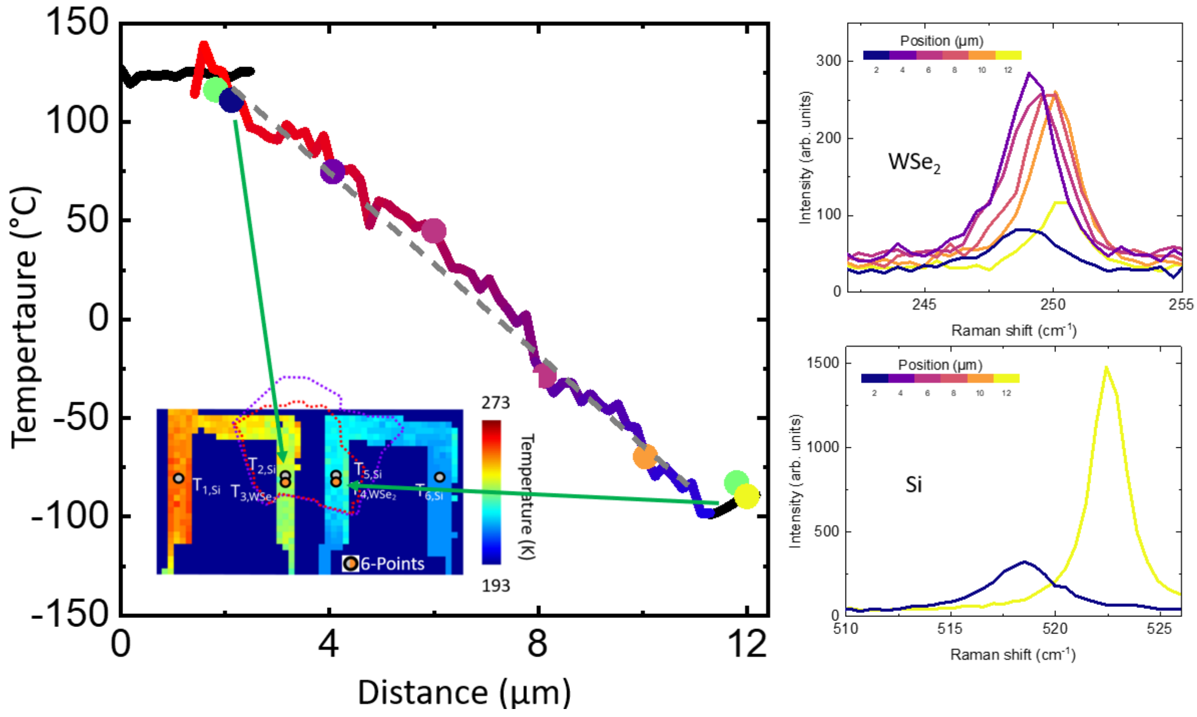

Figure S14-28 The different Raman spectra reported for the sample HS2 for the different materials along the measurement of Figure 4a. The points used for the thermal conductivity are average values obtained at the area localized by the different point in the inset.

15. A reversed temperature bias in Figure 4e

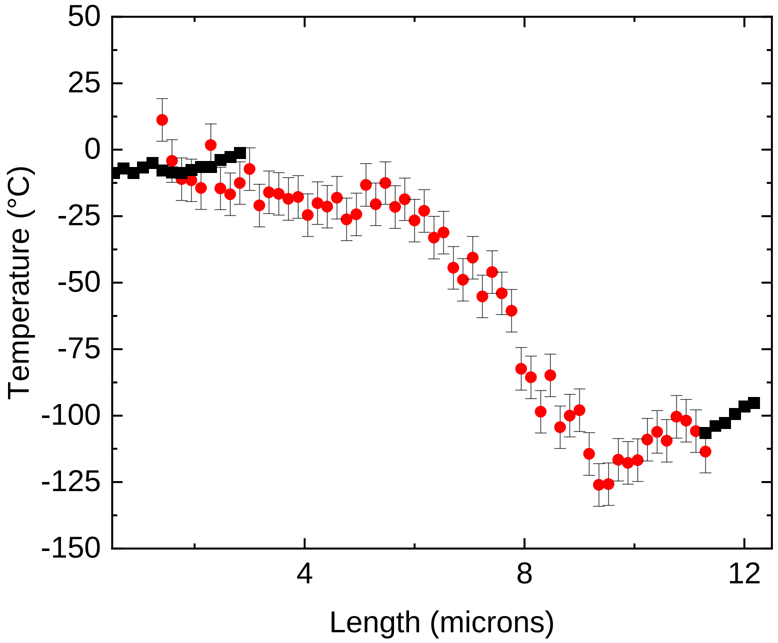

Figure S15-29 An apparent temperature reverse bias within the non-linear temperature profile regime reported for the sample HS2. It is the same data as Figure 4e with uncertainty and more details.

The apparent reversed temperature bias near the cold end in Figure 4e is indeed an intriguing observation that may warrant further investigation if confirmed. At this stage, however, we believe the effect remains within the bounds of experimental uncertainty and cannot be conclusively interpreted unless reproduced consistently. As shown in the expanded plot below, the deviation occurs over only a few data points around x = 10 µm, with an amplitude of roughly two standard deviations, suggesting a small signal close to the measurement noise rather than a genuine temperature inversion.

Regarding the concern about a possible violation of the second law of thermodynamics, we offer the following points for clarification:

The system is not isolated; it is continuously driven out of equilibrium by optical heating at one contact.

Heat transport in such mesoscopic structures involves both forward and backward phonon fluxes, analogous to the Landauer picture for electronic conduction. This highlights that the interpretation of heat flux can be more complex than the macroscopic formulation of the second law and requires considering detailed phonon interactions.

The **apparent local reversal** may simply reflect **complex phonon population dynamics**, involving diverse branches, energies, and momenta, rather than an actual decrease in entropy. In regimes approaching phonon hydrodynamics, simplified models such as the SMA become inadequate.

Related phenomena, including local phonon cooling or parametric squeezing, for example, the one that have been reported in graphene optomechanical systems [101]and other quantum systems. Raman processes themselves can pump or cool specific phonon modes through Stokes and anti-Stokes interactions, illustrating how localized phonon cooling may occur.

Raman thermometry probes primarily the optical phonon population, which may transiently decouple from acoustic phonons in a nonlinear thermal landscape, making a strict thermodynamic interpretation difficult.

A comprehensive understanding of this effect will require dedicated low-temperature experiments and more advanced modeling.

16. Comparison of fitting strategies using different number of sampling points

In general, a two-point configuration is sufficient only when thermal contact resistances are negligible. When the thermal contact resistance becomes comparable to the intrinsic thermal resistance of the sample, at least a four-point measurement is required to properly constrain the temperature profile. In the case of sample HS1, a four-point thermal measurement revealed a small but systematic deviation, of only a few kelvin, between the measured temperature and the simulated temperature map at the coldest region of the silicon microheater (points T1 and T6 in Fig. 3a). This motivated the use of a six-point configuration, which provides a temperature map that is much closer to the experimental reality.
As shown in Figs. 3a–c, the temperature distribution obtained from the six-point analysis already reproduces the experimental data extremely well. Importantly, adding additional sampling points will not lead to any visible improvement in the quality of the fit.

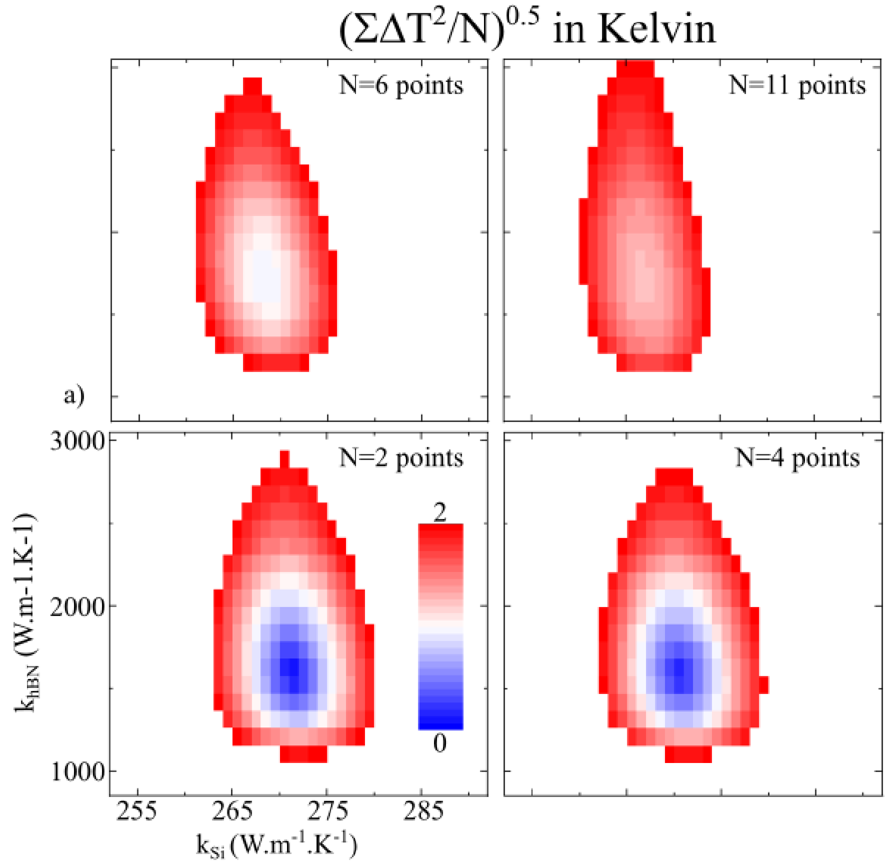

Figure S16: Standard deviation between experimental temperatures and simulated temperature for sample HS1 in function of the hBN thermal conductivity and the Silicon thermal conductivity. We plot the result for N=2,4,6 and 11 points of temperature. The white part corresponds to uncertainty above 2 degrees.

To quantitatively justify this conclusion, we show the standard deviation (in kelvin) between the experimental data and the fitted temperature map as a function of the number of sampling points (N = 2, 4, 6, and 11).
In this analysis, the thermal contact conductance was fixed to the value extracted independently (see Section S11 of the Supplementary Information). This explains why the two-point configuration can still

yield a reasonable result in this specific case, as the contact conductance is not treated as a free parameter. Indeed, the thermal contact conductance cannot be reliably determined from a two-point measurement alone.

We observe that the minimum standard deviation is reached for both the four-point and six-point configurations, with a slightly shift of the minimum standard deviation, i.e the best solution. Between the four-point and six-point analyses, the optimized thermal conductivities of hBN and silicon differ by approximately 100 $W \cdot m^{-1} \cdot K^{-1}$. This difference originates from the improved constraint provided by the additional points located farther from the heater in the six-point configuration and a better fit of our whole data. However, this correction remains relatively small.

A similarly but smaller shift in the optimized parameters is observed when increasing the number of points from six to eleven, again on the order of less than 100 $W \cdot m^{-1} \cdot K^{-1}$ for the hBN thermal conductivity. This seems negligible and this confirms that the six-point configuration already captures the essential physics and provides a robust and accurate fit. Even the 4-point measurements seem accurate from this perspective.

Finally, the apparent increase in the minimum standard deviation with the number of sampling points is a generic fitting effect: while a two-point dataset can always be fitted perfectly, this is no longer true

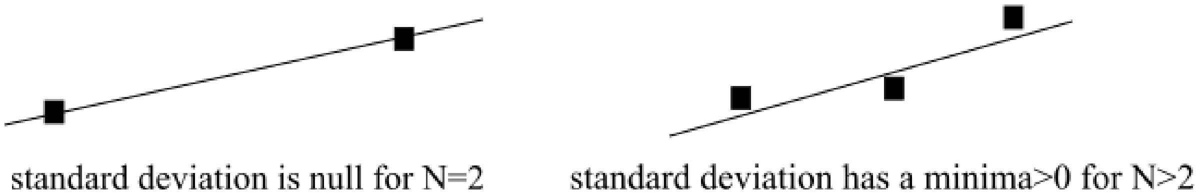

when fitting three or more points, as can be explained schematically on the Figure below. This behavior is analogous to fitting a straight line through two points versus three or more points.

Based on this systematic analysis, we conclude that using more than six sampling points does not significantly improve the fitting quality, while increasing experimental and computational complexity. The six-point configuration therefore represents an optimal compromise between accuracy and efficiency.

Supplementary references

1. Pizzocchero, F. *et al.* The hot pick-up technique for batch assembly of van der Waals heterostructures. *Nat. Commun.* **7**, 11894 (2016).
2. Wang, L. *et al.* One-Dimensional Electrical Contact to a Two-Dimensional Material. *Science* **342**, 614–617 (2013).
3. Jia, H. *et al.* Large-scale arrays of single- and few-layer MoS2 nanomechanical resonators. *Nanoscale* **8**, 10677–10685 (2016).
4. Ma, X. *et al.* Capillary-Force-Assisted Clean-Stamp Transfer of Two-Dimensional Materials. *Nano Lett.* **17**, 6961–6967 (2017).
5. Chiout, A. *et al.* Extreme mechanical tunability in suspended MoS2 resonator controlled by Joule heating. *Npj 2D Mater. Appl.* **7**, 1–8 (2023).
6. Chiout, A. *et al.* High Strain Engineering of a Suspended WSSe Monolayer Membrane by Indentation and Measured by Tip-Enhanced Photoluminescence. *Adv. Opt. Mater.* **12**, 2302369 (2024).
7. Chiout, A., Brochard-Richard, C., Oehler, F., Ouerghi, A. & Chaste, J. Mapping of Vibrational Modes Revealing a Strong and Tunable Coupling in Two Juxtaposed 3R-WSe2 Nanodrums. *Nano Lett.* **24**, 10148–10154 (2024).
8. dos Santos, E. P. *et al.* Temperature dependence of the double-resonance Raman bands in bilayer WSe2. *Vib. Spectrosc.* **110**, 103117 (2020).

9. Verma, P., Abbi, S. & Jain, K. Raman-Scattering Probe of Anharmonic Effects in Gaas. *Phys. Rev. B* **51**, 16660–16667 (1995).
10. Balkanski, M., Wallis, R. & Haro, E. Anharmonic Effects in Light-Scattering Due to Optical Phonons in Silicon. *Phys. Rev. B* **28**, 1928–1934 (1983).
11. Sahoo, S., Gaur, A. P. S., Ahmadi, M., Guinel, M. J.-F. & Katiyar, R. S. Temperature-Dependent Raman Studies and Thermal Conductivity of Few-Layer MoS_2. *J. Phys. Chem. C* **117**, 9042–9047 (2013).
12. Taube, A. *et al.* Temperature-Dependent Nonlinear Phonon Shifts in a Supported MoS_2 Monolayer. *ACS Appl. Mater. INTERFACES* **6**, 8959–8963 (2014).
13. Taube, A., Judek, J., Lapiriska, A. & Zdrojek, M. Temperature-Dependent Thermal Properties of Supported MoS_2 Monolayers. *ACS Appl. Mater. INTERFACES* **7**, 5061–5065 (2015).
14. Lanzillo, N. A. *et al.* Temperature-dependent phonon shifts in monolayer MoS_2. *Appl. Phys. Lett.* **103**, 093102 (2013).
15. Li, Z. *et al.* Temperature-dependent Raman spectroscopy studies of 1-5-layer WSe_2. *NANO Res.* **13**, 591–595 (2020).
16. Balandin, A. A. *et al.* Superior Thermal Conductivity of Single-Layer Graphene. *Nano Lett.* **8**, 902–907 (2008).
17. Chaste, J. *et al.* Nanomechanical Strain Concentration on a Two-Dimensional Nanobridge within a Large Suspended Bilayer Graphene for Molecular Mass Detection. *ACS Appl. Nano Mater.* **1**, 6752–6759 (2018).
18. Li, X. *et al.* Temperature Dependence of Raman-Active In-Plane E2g Phonons in Layered Graphene and h-BN Flakes. *Nanoscale Res. Lett.* **13**, 25 (2018).
19. Cuscó, R. *et al.* Isotopic effects on phonon anharmonicity in layered van der Waals crystals: Isotopically pure hexagonal boron nitride. *Phys. Rev. B* **97**, 155435 (2018).
20. Stenger, I. *et al.* Low frequency Raman spectroscopy of few-atomic-layer thick hBN crystals. *2D Mater.* **4**, 031003 (2017).
21. Cuscó, R., Gil, B., Cassabois, G. & Artús, L. Temperature dependence of Raman-active phonons and anharmonic interactions in layered hexagonal BN. *Phys. Rev. B* **94**, 155435 (2016).
22. Zhou, H. *et al.* High thermal conductivity of suspended few-layer hexagonal boron nitride sheets. *Nano Res.* **7**, 1232–1240 (2014).
23. Cai, Q. *et al.* High thermal conductivity of high-quality monolayer boron nitride and its thermal expansion. *Sci. Adv.* **5**, eaav0129 (2019).
24. Mercado, E. *et al.* Isotopically Enhanced Thermal Conductivity in Few-Layer Hexagonal Boron Nitride: Implications for Thermal Management. *ACS Appl. Nano Mater.* **3**, 12148–12156 (2020).
25. Tsu, R. & Hernandez, J. G. Temperature dependence of silicon Raman lines. *Appl. Phys. Lett.* **41**, 1016–1018 (1982).
26. Hart, T. R., Aggarwal, R. L. & Lax, B. Temperature Dependence of Raman Scattering in Silicon. *Phys. Rev. B* **1**, 638–642 (1970).
27. Okada, Y. & Tokumaru, Y. Precise determination of lattice parameter and thermal expansion coefficient of silicon between 300 and 1500 K. *J. Appl. Phys.* **56**, 314–320 (1984).
28. Tang, X., Yue, Y., Chen, X. & Wang, X. Sub-wavelength temperature probing in near-field laser heating by particles. *Opt. Express* **20**, 14152–14167 (2012).
29. Périchon, S., Lysenko, V., Remaki, B., Barbier, D. & Champagnon, B. Measurement of porous silicon thermal conductivity by micro-Raman scattering. *J. Appl. Phys.* **86**, 4700–4702 (1999).
30. Tuschel, D. & Adar, F. Molecular Spectroscopy Workbench Raman Thermometry. *SPECTROSCOPY* **31**, 8–13 (2016).
31. Tuschel, D. Raman Thermometry: Understanding the Mathematics to Better Design Raman Measurements. *SPECTROSCOPY* **34**, 8–13 (2019).
32. Fujimori, H., Kakihana, M., Ioku, K., Goto, S. & Yoshimura, M. Advantage of anti-Stokes Raman scattering for high-temperature measurements. *Appl. Phys. Lett.* **79**, 937–939 (2001).
33. Long, D. A. (Derek A. *Raman Spectroscopy*. (New York : McGraw-Hill, 1977).
34. Hayes, W. & Loudon, R. *Scattering of Light by Crystals*. (Wiley, 1978).

35. McGrane, S. D., Moore, D. S., Goodwin, P. M. & Dattelbaum, D. M. Quantitative Tradeoffs between Spatial, Temporal, and Thermometric Resolution of Nonresonant Raman Thermometry for Dynamic Experiments. *Appl. Spectrosc.* **68**, 1279–1288 (2014).
36. Jesus Gallardo, J. *et al.* Micro-Raman Spectroscopy for the Determination of Local Temperature Increases in TiO_2 Thin Films due to the Effect of Radiation. *Appl. Spectrosc.* **70**, 1128–1136 (2016).
37. Kip, B. & Meier, R. Determination of the Local Temperature at a Sample During Raman Experiments Using Stokes and Anti-Stokes Raman Bands. *Appl. Spectrosc.* **44**, 707–711 (1990).
38. Blundo, E. *et al.* Vibrational Properties in Highly Strained Hexagonal Boron Nitride Bubbles. *Nano Lett.* **22**, 1525–1533 (2022).
39. Glassbrenner, C. J. & Slack, G. A. Thermal Conductivity of Silicon and Germanium from 3°K to the Melting Point. *Phys. Rev.* **134**, A1058–A1069 (1964).
40. Fulkerson, W., Moore, J. P., Williams, R. K., Graves, R. S. & McElroy, D. L. Thermal Conductivity, Electrical Resistivity, and Seebeck Coefficient of Silicon from 100 to 1300°K. *Phys. Rev.* **167**, 765–782 (1968).
41. Shanks, H. R., Maycock, P. D., Sidles, P. H. & Danielson, G. C. Thermal Conductivity of Silicon from 300 to 1400°K. *Phys. Rev.* **130**, 1743–1748 (1963).
42. Yuan, C. *et al.* Modulating the thermal conductivity in hexagonal boron nitride via controlled boron isotope concentration. *Commun. Phys.* **2**, 43 (2019).
43. Thi, T. T. N. Numerical study of electro-thermal effects in silicon devices.
44. Jiang, P., Qian, X., Gu, X. & Yang, R. Probing Anisotropic Thermal Conductivity of Transition Metal Dichalcogenides MX2 (M = Mo, W and X = S, Se) using Time-Domain Thermoreflectance. *Adv. Mater.* **29**, 1701068 (2017).
45. Liu, J., Choi, G.-M. & Cahill, D. G. Measurement of the anisotropic thermal conductivity of molybdenum disulfide by the time-resolved magneto-optic Kerr effect. *J. Appl. Phys.* **116**, 233107 (2014).
46. Zhang, X. *et al.* Measurement of Lateral and Interfacial Thermal Conductivity of Single- and Bilayer MoS2 and MoSe2 Using Refined Optothermal Raman Technique. *ACS Appl. Mater. Interfaces* **7**, 25923–25929 (2015).
47. Gu, X., Li, B. & Yang, R. Layer thickness-dependent phonon properties and thermal conductivity of MoS2. *J. Appl. Phys.* **119**, 085106 (2016).
48. Pisoni, A. *et al.* Anisotropic transport properties of tungsten disulfide. *Scr. Mater.* **114**, 48–50 (2016).
49. Jaffe, G. R. *et al.* Thickness-Dependent Cross-Plane Thermal Conductivity Measurements of Exfoliated Hexagonal Boron Nitride. *ACS Appl. Mater. Interfaces* **15**, 12545–12550 (2023).
50. A Simpson & A D Stuckes. The thermal conductivity of highly oriented pyrolytic boron nitride. *J. Phys. C Solid State Phys.* **4**, 1710 (1971).
51. Jiang, P., Qian, X. & Yang, R. Time-domain thermoreflectance (TDTR) measurements of anisotropic thermal conductivity using a variable spot size approach. *Rev. Sci. Instrum.* **88**, 074901 (2017).
52. Jiang, P., Qian, X., Yang, R. & Lindsay, L. Anisotropic thermal transport in bulk hexagonal boron nitride. *Phys. Rev. Mater.* (2018).
53. Gabourie, A. J., Suryavanshi, S. V., Farimani, A. B. & Pop, E. Reduced thermal conductivity of supported and encased monolayer and bilayer MoS2. *2D Mater.* **8**, 011001 (2020).
54. Cai, W. *et al.* Thermal Transport in Suspended and Supported Monolayer Graphene Grown by Chemical Vapor Deposition. *Nano Lett.* **10**, 1645–1651 (2010).
55. Chen, J., Zhang, G. & Li, B. Substrate coupling suppresses size dependence of thermal conductivity in supported graphene. *Nanoscale* **5**, 532–536 (2012).
56. Sang, Y. *et al.* Measurement of Thermal Conductivity of Suspended and Supported Single-Layer WS2 Using Micro-photoluminescence Spectroscopy. *J. Phys. Chem. C* **126**, 6637–6645 (2022).
57. Chen, S. *et al.* Thermal conductivity of isotopically modified graphene. *Nat. Mater.* **11**, 203–207 (2012).

58. Rahimi, M. *et al.* Complete Determination of Thermoelectric and Thermal Properties of Supported Few-Layer Two-Dimensional Materials. *Phys. Rev. Appl.* **19**, 034075 (2023).
59. Sichel, E. K., Miller, R. E., Abrahams, M. S. & Buiocchi, C. J. Heat capacity and thermal conductivity of hexagonal pyrolytic boron nitride. *Phys. Rev. B* **13**, 4607–4611 (1976).
60. Rahman, M., Shahzadeh, M. & Pisana, S. Simultaneous measurement of anisotropic thermal conductivity and thermal boundary conductance of 2-dimensional materials. *J. Appl. Phys.* **126**, 205103 (2019).
61. Cai, Q. *et al.* Outstanding Thermal Conductivity of Single Atomic Layer Isotope-Modified Boron Nitride. *Phys. Rev. Lett.* **125**, 085902 (2020).
62. Wang, C. *et al.* Superior thermal conductivity in suspended bilayer hexagonal boron nitride. *Sci. Rep.* **6**, 25334 (2016).
63. Jo, I. *et al.* Thermal Conductivity and Phonon Transport in Suspended Few-Layer Hexagonal Boron Nitride. *Nano Lett.* **13**, 550–554 (2013).
64. Chang, C. W. *et al.* Isotope Effect on the Thermal Conductivity of Boron Nitride Nanotubes. *Phys. Rev. Lett.* **97**, 085901 (2006).
65. Ouyang, T. *et al.* Thermal transport in hexagonal boron nitride nanoribbons. *Nanotechnology* **21**, 245701 (2010).
66. Lindsay, L. & Broido, D. A. Theory of thermal transport in multilayer hexagonal boron nitride and nanotubes. *Phys. Rev. B* **85**, 035436 (2012).
67. Illera, S., Pruneda, M., Colombo, L. & Ordejón, P. Thermal and transport properties of pristine single-layer hexagonal boron nitride: A first principles investigation. *Phys. Rev. Mater.* (2017).
68. Dong, H., Hirvonen, P., Fan, Z. & Ala-Nissila, T. Heat transport in pristine and polycrystalline single-layer hexagonal boron nitride. *Phys. Chem. Chem. Phys.* **20**, 24602–24612 (2018).
69. Lindsay, L. & Broido, D. A. Enhanced thermal conductivity and isotope effect in single-layer hexagonal boron nitride. *Phys. Rev. B* (2011).
70. Rahman, Md. H. *et al.* Phonon thermal conductivity of the stanene/hBN van der Waals heterostructure. *Phys. Chem. Chem. Phys.* **23**, 11028–11038 (2021).
71. Sevik, C., Kinaci, A., Haskins, J. B. & Çağın, T. Influence of disorder on thermal transport properties of boron nitride nanostructures. *Phys. Rev. B* **86**, 075403 (2012).
72. Klemens', P. G. & Pedraza, D. F. THERMAL CONDUCTIVITY OF GRAPHITE IN THE BASAL PLANE.
73. Jo, I. *et al.* Reexamination of basal plane thermal conductivity of suspended graphene samples measured by electro-thermal micro-bridge methods. *AIP Adv.* **5**, 053206 (2015).
74. Sadeghi, M. M., Jo, I. & Shi, L. Phonon-interface scattering in multilayer graphene on an amorphous support. *Proc. Natl. Acad. Sci.* **110**, 16321–16326 (2013).
75. Jang, W., Chen, Z., Bao, W., Lau, C. N. & Dames, C. Thickness-Dependent Thermal Conductivity of Encased Graphene and Ultrathin Graphite. *Nano Lett.* **10**, 3909–3913 (2010).
76. Li, Q.-Y. *et al.* Measurement of specific heat and thermal conductivity of supported and suspended graphene by a comprehensive Raman optothermal method. *Nanoscale* **9**, 10784–10793 (2017).
77. Yang, J. *et al.* Thermal conductance imaging of graphene contacts. *J. Appl. Phys.* **116**, 023515 (2014).
78. Seol, J. H. *et al.* Two-Dimensional Phonon Transport in Supported Graphene. *Science* **328**, 213–216 (2010).
79. Liu, J. *et al.* Thermal conductivity of giant mono- to few-layered CVD graphene supported on an organic substrate. *Nanoscale* **8**, 10298–10309 (2016).
80. Judek, J. *et al.* High accuracy determination of the thermal properties of supported 2D materials. *Sci. Rep.* **5**, 12422 (2015).
81. Machida, Y., Matsumoto, N., Isono, T. & Behnia, K. Phonon hydrodynamics and ultrahigh–room-temperature thermal conductivity in thin graphite. *Science* **367**, 309–312 (2020).
82. Li, H. *et al.* Thermal conductivity of twisted bilayer graphene. *Nanoscale* **6**, 13402–13408 (2014).

83. Chen, S. *et al.* Raman Measurements of Thermal Transport in Suspended Monolayer Graphene of Variable Sizes in Vacuum and Gaseous Environments. *ACS Nano* **5**, 321–328 (2011).
84. Chen, S. *et al.* Thermal conductivity measurements of suspended graphene with and without wrinkles by micro-Raman mapping. *Nanotechnology* **23**, 365701 (2012).
85. Lee, J.-U., Yoon, D., Kim, H., Lee, S. W. & Cheong, H. Thermal conductivity of suspended pristine graphene measured by Raman spectroscopy. *Phys. Rev. B* (2011).
86. Malekpour, H. *et al.* Thermal conductivity of graphene with defects induced by electron beam irradiation. *Nanoscale* **8**, 14608–14616 (2016).
87. Faugeras, C. *et al.* Thermal Conductivity of Graphene in Corbino Membrane Geometry. *ACS Nano* **4**, 1889–1892 (2010).
88. Fugallo, G. *et al.* Thermal Conductivity of Graphene and Graphite: Collective Excitations and Mean Free Paths. *Nano Lett.* **14**, 6109–6114 (2014).
89. Saleta Reig, D. *et al.* Unraveling Heat Transport and Dissipation in Suspended MoSe2 from Bulk to Monolayer. *Adv. Mater.* **34**, 2108352 (2022).
90. Pisoni, A. *et al.* The Role of Transport Agents in MoS2 Single Crystals. *J. Phys. Chem. C* **119**, 3918–3922 (2015).
91. Yan, R. *et al.* Thermal Conductivity of Monolayer Molybdenum Disulfide Obtained from Temperature-Dependent Raman Spectroscopy. *ACS Nano* **8**, 986–993 (2014).
92. Jo, I., Pettes, M. T., Ou, E., Wu, W. & Shi, L. Basal-plane thermal conductivity of few-layer molybdenum disulfide. *Appl. Phys. Lett.* **104**, 201902 (2014).
93. Yu, Y., Ibn Minhaj, T., Huang, L., Yu, Y. & Cao, L. In-Plane and Interfacial Thermal Conduction of Two-Dimensional Transition-Metal Dichalcogenides. *Phys. Rev. Appl.* **13**, (2020).
94. Bae, J. J. *et al.* Thickness-dependent in-plane thermal conductivity of suspended MoS2 grown by chemical vapor deposition. *Nanoscale* **9**, 2541–2547 (2017).
95. Morell, N. *et al.* Optomechanical Measurement of Thermal Transport in Two-Dimensional MoSe2 Lattices. *Nano Lett.* **19**, 3143–3150 (2019).
96. Chen, K. *et al.* Ultrahigh thermal conductivity in isotope-enriched cubic boron nitride. *Science* **367**, 555–559 (2020).
97. Inyushkin, A. V. Thermal conductivity of high purity synthetic single crystal diamonds. *Phys. Rev. B* (2018).
98. Cepellotti, A. *et al.* Phonon hydrodynamics in two-dimensional materials. *Nat. Commun.* **6**, 6400 (2015).
99. Fournier, D., Marangolo, M. & Fretigny, C. Measurement of thermal properties of bulk materials and thin films by modulated thermoreflectance (MTR). *J. Appl. Phys.* **128**, 241101 (2020).
100. Rahimi, M. *et al.* Complete Determination of Thermoelectric and Thermal Properties of Supported Few-Layer Two-Dimensional Materials. *Phys. Rev. Appl.* **19**, 034075 (2023).
101. Barton, R. A. *et al.* Photothermal Self-Oscillation and Laser Cooling of Graphene Optomechanical Systems. *Nano Lett.* **12**, 4681–4686 (2012).
